# Co-occurrence of Mild Salinity and Drought Synergistically Enhances Biomass and Grain Retardation in Wheat

**DOI:** 10.3389/fpls.2019.00501

**Published:** 2019-04-24

**Authors:** Kenny Paul, János Pauk, Ankica Kondic-Spika, Heinrich Grausgruber, Tofig Allahverdiyev, László Sass, Imre Vass

**Affiliations:** ^1^Institute of Plant Biology, Biological Research Centre, Hungarian Academy of Sciences, Szeged, Hungary; ^2^Department of Biotechnology, Cereal Research Non-Profit Ltd., Szeged, Hungary; ^3^Institute of Field and Vegetable Crops, Novi Sad, Serbia; ^4^Department of Crop Sciences, University of Natural Resources and Life Sciences, Vienna, Austria; ^5^Research Institute of Crop Husbandry, Ministry of Agriculture of Azerbaijan Republic, Baku, Azerbaijan; ^6^Institute of Molecular Biology and Biotechnology, Azerbaijan National Academy of Sciences, Baku, Azerbaijan

**Keywords:** drought stress, salt stress, interaction of drought and salt stress, high throughput phenotyping, wheat

## Abstract

In the present study we analyzed the responses of wheat to mild salinity and drought with special emphasis on the so far unclarified interaction of these important stress factors by using high-throughput phenotyping approaches. Measurements were performed on 14 genotypes of different geographic origin (Austria, Azerbaijan, and Serbia). The data obtained by non-invasive digital RGB imaging of leaf/shoot area reflect well the differences in total biomass measured at the end of the cultivation period demonstrating that leaf/shoot imaging can be reliably used to predict biomass differences among different cultivars and stress conditions. On the other hand, the leaf/shoot area has only a limited potential to predict grain yield. Comparison of gas exchange parameters with biomass accumulation showed that suppression of CO_2_ fixation due to stomatal closure is the principal cause behind decreased biomass accumulation under drought, salt and drought plus salt stresses. Correlation between grain yield and dry biomass is tighter when salt- and drought stress occur simultaneously than in the well-watered control, or in the presence of only salinity or drought, showing that natural variation of biomass partitioning to grains is suppressed by severe stress conditions. Comparison of yield data show that higher biomass and grain yield can be expected under salt (and salt plus drought) stress from those cultivars which have high yield parameters when exposed to drought stress alone. However, relative yield tolerance under drought stress is not a good indicator of yield tolerance under salt (and salt plus drought) drought stress. Harvest index of the studied cultivars ranged between 0.38 and 0.57 under well watered conditions and decreased only to a small extent (0.37–0.55) even when total biomass was decreased by 90% under the combined salt plus drought stress. It is concluded that the co-occurrence of mild salinity and drought can induce large biomass and grain yield losses in wheat due to synergistic interaction of these important stress factors. We could also identify wheat cultivars, which show high yield parameters under the combined effects of salinity and drought demonstrating the potential of complex plant phenotyping in breeding for drought and salinity stress tolerance in crop plants.

## Introduction

Drought and salinity are two widespread environmental abiotic stress factors in many regions. Soil salinization is one of the serious forms of soil degradation, which can arise from natural causes and human-mediated activity, such as irrigation in arid and semi-arid regions (Rengasamy et al., 2010). More than 800 million hectares of land throughout the world are salt-affected, which has important consequences for the productivity of wheat and other crops. Increased soil salt concentrations decrease the ability of plants to take up water leading to apparent water limitation, or can lead to the accumulation of salt in the shoots, which negatively affects growth by impairing metabolic processes and decreasing photosynthetic efficiency, partly through stomatal closure (Flowers and Yeo, 1995; Maser et al., 2002; Munns, 2002; Roy et al., 2014). As a consequence of the ongoing global climate changes low water availability and salinization are expected to affect up to 50% of all arable lands by the year 2050 (Wang et al., 2003), which will hamper efforts to meet the dramatically increasing demand for food predicted by the same year (Cobb et al., 2013). Salinity can affect plant functions via two main mechanisms (Munns and James, 2003; Munns and Tester, 2008; Arzani and Ashraf, 2016): (i) via inducing external osmotic pressure around the roots in the soil, which decreases the uptake of water leading symptoms similar to caused by drought, and (ii) via toxic effect of salt ions, mostly Na^+^ and Cl^-^, which accumulate in the plant tissues, mostly in the leaves. The osmotic effect is characteristic at low level of salinity and in the initial phases of salt exposure, while the ionic effect occurs during long term exposure and at high levels of salinity (Munns and James, 2003; Munns and Tester, 2008; Arzani and Ashraf, 2016).

Drought is one of the most common environmental stresses that affect growth and development of plants, and continues to be an important challenge to agricultural researchers and plant breeders. It induces a shortage of water in the root zone resulting in osmotic imbalance leading to decreased yield (Salekdeh et al., 2009). Plants can survive drought by adaptive morphological and physiological changes, which are reflected in their biochemical processes. Drought stress often induces stomatal closure that restricts the diffusion of CO_2_ into the leaf or due to non-stomatal limitations, which leads to decrease of carbon assimilation and other processes of photosynthesis (Ashraf and Harris, 2013; Guan et al., 2015; Paul et al., 2016). Since salinity decreases water uptake through the roots the co-occurrence of water shortage and saline conditions can yield serious stress conditions.

Climate changes during last three decades have already caused a significant yield loss and the global production of wheat was decreased by 5.5% (Lobell et al., 2011). Global environmental changes are expected to continue causing increasing occurrence and severity of both drought and salt stresses (Tester and Langridge, 2010), which will impact further food availability. Therefore, breeding of crops for stress tolerance, including drought and salinity, plays a significant role in agriculture and requires proper understanding of physiological characteristics and natural variations.

These data highlight the importance of studies addressing stress physiology and spurred research on understanding the mechanisms plants activate to respond to water, salt and other abiotic stresses (Kumar et al., 2017). Physiological responses of plants to drought and salinity stress have common features. Both stresses induce cellular dehydration, which causes osmotic stress and removal of water from the cytoplasm into the intercellular space leading to stomatal closure, which affects CO_2_ fixation, etc. (Flexas et al., 2004, 2007). Individually, salt and drought stress conditions have been the subject of intense research (Nevo and Chen, 2010; Zhang et al., 2014; Landi et al., 2017). However, in the field plants are often exposed to the combination of two or more stresses. Studies dealing with the combination of drought and heat demonstrated that responses to combined stresses cannot be simply extrapolated from the responses of plants to these different stresses when applied individually, and this is expected to be case for salinity and drought (see Mittler, 2006). The specific effect of combined salt- and drought stress on wheat plants has been the topic of only few previous studies, which indicated that salinity and drought can interact and may enhance or even ameliorate each other’s effects (Chen et al., 2009; Dugasa et al., 2018). However, these studies compared only limited number (2) of cultivars and did not address the complex response of biomass, grain yield, water usage and photosynthetic parameters in detail.

High throughput phenotyping approaches are rapidly gaining popularity in tracking morphological and physiological changes of plants under stress conditions, where digital color imaging can be used to quantify plant biomass, leaf area and plant height (Majer et al., 2008; Rajendram et al., 2009; Harris et al., 2010; Fehér-Juhász et al., 2014; Ghanem et al., 2015; Al-Tamini et al., 2016). These parameters in combination with photosynthetic activity measurements can provide valuable information on the extent and mechanisms of stress induced crop yield loss, which can be utilized in the selection of tolerant cultivars.

In the present work we have studied the interacting effects of mild salinity and drought stress in bread wheat (*Triticum aestivum* L.), which is a highly important crop with moderate tolerance against salinity and drought (Munns and Tester, 2008). The basic question of our work was to test if growth and grain production in wheat can be affected, either positively or negatively, by the co-occurrence of mild salinity and drought, which conditions are expected to be more common in the future due to the ongoing climate changes. Seedlings of wheat cultivars originating from Austria, Azerbaijan, and Serbia were monitored during their whole life cycle using the combination of phenotyping and photosynthetic tools. An assessment was made of the interacting effects of drought and salinity on biomass accumulation and grain yield, as well as on photosynthetic electron transport, net gas exchange, and antioxidant compounds. The data show that the co-occurring saline and drought conditions synergistically interact and induce higher loss of photosynthetic and yield parameters than would be expected if these two stress factors are acted independently from each other.

## Materials and Methods

### Plant Material and Experimental Details

The experiments were conducted with 14 wheat (*Triticum aestivum* L.) cultivars from Serbia (5), Austria (4), and Azerbaijan (5), which were chosen on the basis of preliminary data available for their drought tolerance (Table 1). Vernalization of 1-week-old seedlings was carried out for 6 weeks, at 4°C in a cold chamber, under continuous dim light (50 μmol photons m^-2^ s^-1^ light intensity). Vernalized plantlets were planted into plastic pots containing the mixture of Terra peat soil and sandy soil (3:1, v/v). Equal amount of fertilizer (SUBSTRAL^®^ Osmocote Plus^®^ containing 15% N, 10% P, 12% K, 2% Mg, supplemented with microelements 0.02% B, 0.05% Cu, 0.4% Fe, 0.06% Mn, 0.02% Mo, 0.015% Zn) was added to each pot (2.8 g/l fertilizer in 1870 g soil mixture). Pot volume was 2 L with 13 cm diameter. Each pot contained only one plants. Plants were regularly irrigated and grown in controlled green-house conditions for 2 weeks before starting the stress treatments. Photosynthetically active radiation (PAR) within the controlled environment was maintained with a 14 h photoperiod at a PPFD of 400 – 500 μmol photons m^-2^ s^-1^, 22–25°C and ca. 45–55% relative humidity.

**Table 1:** Literature information of wheat cultivars used in the study.

Country of origin	Name	Drought tolerance	References
Austria	‘Donnato’		
Austria	‘Midas’	Sensitive	Teizer, 2010
Austria	‘Gallio’		
Austria	‘Capo’	Tolerant	Teizer, 2010
Azerbaijan	‘Tale 38’	Sensitive	Talai, 2010
Azerbaijan	‘Azamatli 95’	Tolerant	Huseynova et al., 2007; Talai, 2010
Azerbaijan	‘Giymatli 2/17’	Sensitive	Huseynova et al., 2007
Azerbaijan	‘Gobustan’	Tolerant	Talai, 2010
Azerbaijan	‘Gyrmyzy gul- 1’	Sensitive	Talai, 2010; Babayev et al., 2013
Serbia	‘Balkan’		
Serbia	‘NS 40S’	Tolerant	Babic et al., 2011
Serbia	‘NS Avangarda’		
Serbia	‘Suboticanka’	Sensitive	Dencic et al., 2000
Serbia	‘Renesansa’		Dimitrijevic et al., 2009

Plants were grown under four different water/salt treatment (T) conditions:

(i) T1, well watered (60% soil water capacity) and no salt (NaCl) added,
(ii) T2, water limited (20% soil water capacity) and no salt (NaCl) added,
(iii) T3, well watered (60% soil water capacity) and saline conditions (0.2% NaCl, i.e., 2 g /kg soil), and
(iv) T4, water limited (20% soil water capacity) and saline conditions (0.2% NaCl, i.e., 2 g /kg soil).

The water retention curve (Supplementary Figure 1 and Supplementary Table 1) shows that the well watered (T1, T3) and water limited (T2, T4) conditions correspond to -3 and -3500 kPa soil water potential, respectively.

Salt was mixed in the soil before planting the seedlings in order to ensure that plants are exposed to the same level of salinity during their whole growth. Pots were closed on the bottom, so the salt was not washed out from the soil during the experiments. Controlled watering conditions were ensured by using the computer-controlled water supply system of our plant phenotyping platform (Cseri et al., 2013; Fehér-Juhász et al., 2014). All plants were individually identified with radio frequency ID tags, and were watered according to the preset watering protocols, in order to maintain 20 or 60% soil water capacity, under water limited and well watered conditions, respectively. When the peat-sand mix is filled in the pots the soil water capacity is ca. 40%. At planting the seedlings 150 mL water is added to each pot containing 1870 g soil. The first adjustment water content was after 1 (well watered)-2 (water limited) days to keep plants growing. In case of the well watered (T1, T3) plants computer assisted water adjustment was done every 2–3 days to keep the target 60% soil water capacity. In case of the water limited plants (T2, T4) the soil reached the 20% water capacity in ca. 1 week after which this level was maintained by watering at ca. once a week. Water use profiles were recorded at the level of individual plants during the whole cultivation period from which the efficiency of water usage, as well as the effect of NaCl on water utilization was determined. Five replicates of each treatment were used for the experimental studies conducted for 4 months (February–May) at the greenhouse of the Cereal Research Ltd., Szeged, Hungary.

### Biomass and Grain Yield Estimation

The shoot growth parameters were analyzed by digital RGB imaging, which makes possible to separate plants from their environment, using the HAS-SSDS phenotyping platform as described earlier (Cseri et al., 2013; Fehér-Juhász et al., 2014). These measurements provided information on plant height, and total green biomass change during the whole cultivation period. At the end of the experiment (13 weeks after the stress treatments were started) grain production parameters (above ground biomass, plant height, number of spikelet and seed per spike, total grain weight) were determined by traditional methods. From the ratio of the grain yield and above ground dry biomass the harvest index (HI) can be calculated.

### Gas Exchange Measurements

Gas exchange parameters: CO_2_ uptake rate, transpiration, stomatal conductance and intercellular CO_2_ concentration were measured by using a Licor 6400 gas analyzer (Licor, United States). Two to three selected pieces of attached leaves from plant replicates under respective treatments were inserted into the leaf chamber (6 cm^2^) for individual measurements (Paul et al., 2016). The gas cuvette conditions were set to 400 ppm CO_2_, ambient temperature and growth light intensity of photosynthetic active radiation (400 μmol photons m^-2^ s^-1^).

### Electron Transport Rate of Photosystem II (ETR II)

ETR(II) was monitored by using a Mini PAM photosynthesis yield analyzer (WALZ, Effeltrich, Germany) in the reproductive grain filling phase. The measurements were performed on the flag leaf (Paul et al., 2016) in the 6th and 7th week after the start of the stress treatments. The apparent rate of electron transport was calculated as ETR(II) = Y(II) × PPFD × 0.5 × 0.84 (Genty et al., 1989), where Y(II) is the effective quantum yield of PSII, PPFD is the photon flux density of incident PAR. The two coefficients (0.5 and 0.84) represent the fraction of absorbed light partitioned to PSII, and the probability that the incident irradiance will be absorbed by PSII in higher plants, respectively (Björkman and Demmig, 1987; Schreiber, 2004).

### Proline Content Determination

Fresh leaf samples (0.1 g from the fully developed leaf below the flag leaf) were collected from all studied wheat cultivars and stored in liquid nitrogen. The content of free proline was determined as described earlier (Bates et al., 1973) at the 10th week after the start of the stress treatments. Samples were homogenized in 3% (w/v) sulfosalicylic acid to precipitate protein, and centrifuged at 14,000 × *g* for 10 min. The reaction mixture contained 2 mL glacial acetic acid, 2 mL ninhydrin reagent (2.50% w/v ninhydrin in 60 % v/v 6 M phosphoric acid) and 2 mL of the supernatant. The incubation lasted for 1 h at 90°C then, after stopping the reaction with ice, 4 cm^3^ of toluene was added and vortexed. The upper toluene phase was decanted into a glass cuvette and absorbance was measured at *k* = 520 nm. Each assay was performed in five replicates representing five leaves from different plants for each treatment. The content of proline was expressed as mg g^-1^ fresh weight according to a calibration curve with proline.

### Statistical Analysis

Calculation of mean and SD, tests for normal distribution of data, one-way ANOVA analysis of the significance level between mean differences (Supplementary Tables 3–12), as well as heteroscedasticity tests for the distribution of residuals for the linear regression of data were performed by the XLSTAT-Premium software package (Supplementary Table 2) [Addinsoft (2019), Boston, MA, United States]^1^.

Relative tolerance of biomass and grain yield of any of the cultivars was calculated by expressing the yield parameter (biomass or grain yield) observed under one of the stress conditions (T2, T3, or T4) as a percentage of the same yield parameter of the same cultivar obtained under the well watered (T1) control conditions (Equation 1):

(1) $$\begin{array}{c} {Relative yield tolerance}_{\mathrm{Ti}}(\%)={\mathrm{Yield}}_{\mathrm{Ti}}/{\mathrm{Yield}}_{T1}\times 100 \end{array}$$

where yield stands for either total dry biomass or grain yield, Yield_Ti_ represents the observed yield under one of the stress conditions (T2, T3, or T4 as defined under 2.1), Yield_T1_ is the yield obtained under the well watered (T1) control.

## Results and Discussion

### Effect of Drought and Salt Stress on Green Biomass and Grain Yield

**FIGURE 1:**
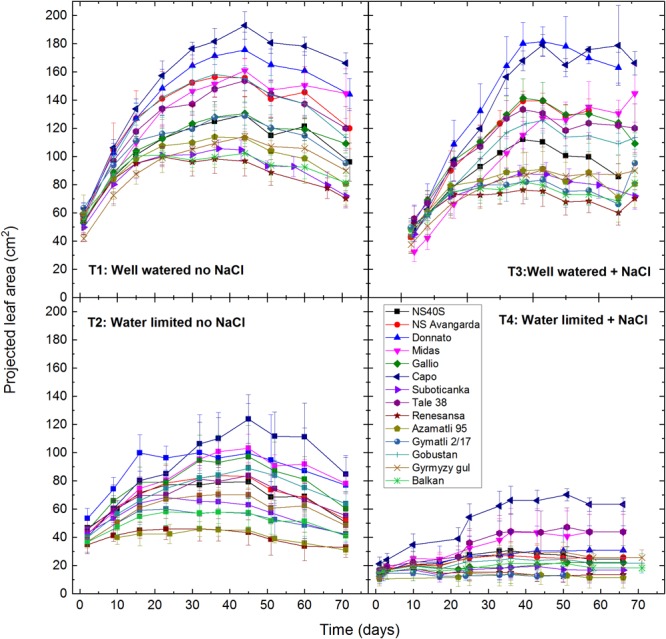
Effect of drought and salt stress on projected leaf area of wheat plants. Digital RGB imaging was used to determine projected green leaf/shoot area of individual plants of the selected 14 cultivars under well watered (T1: 60% soil water capacity), water limited (T2: 20% soil water capacity), well watered plus salt (T3: 2 g NaCl/kg soil, at 60% soil water capacity), and water limited plus salt (T4: 2 g NaCl/kg soil, at 20% soil water capacity) conditions. Data shown are means ± SE (*n* = 5 plants/treatment). Statistical analysis of data is presented in Supplementary Table 3.

Digital imaging is a modern non-invasive method for estimating green biomass of plants on the basis of projected leaf and shoot area (Kacira and Ling, 2001; Furbank and Tester, 2011). Application of this approach has revealed significant variation in the rate of leaf/shoot development under drought and salt stress conditions among the various wheat cultivars used in the present study (Figure 1). Under well watered, T1, conditions variety ‘Capo’ showed the highest projected leaf area reaching its maximum on the 44th day (190 cm^2^), while ‘Renesansa,’ ‘Balkan’ and ‘Suboticanka’ were the lowest (ca. 95 cm^2^). During further cultivation the projected leaf area showed saturation and then gradual decline due to the onset of senescence (Figure 1). Drought stress, T2, drastically reduced the green leaf area in all cultivars (Figure 1). However, ‘Capo’ still showed the highest values at ca. 50% of that observed at well watered conditions, while ‘Renesansa’ and ‘Azamatli 95’ were the most affected by the drought conditions producing only ca. 40% leaf area as compared to the well watered conditions. Green leaf area in general was not significantly affected by mild salt stress under well-watered conditions, T3 (Figure 1). However, there were large cultivar dependent differences in the response to salinity: ‘Capo’ and ‘Donnato’ showed the highest projected leaf/shoot area close to the levels observed in the T1 control. ‘Renesansa’ and ‘Balkan’ produced the lowest green leaf/shoot area, at ca. 75% of the T1 control. When salt was applied together with drought stress, T4, the projected leaf/shoot area decreased drastically in all cultivars. The best performing variety ‘Capo’ decreased the green leaf/shoot area to ca. 30% of its T1 control (Figure 1). In the extreme case of ‘Azamatli 95’ ca. 84% leaf/shoot area loss was observed under the combination of salinity and drought relative to T1 (Figure 1).

**FIGURE 2:**
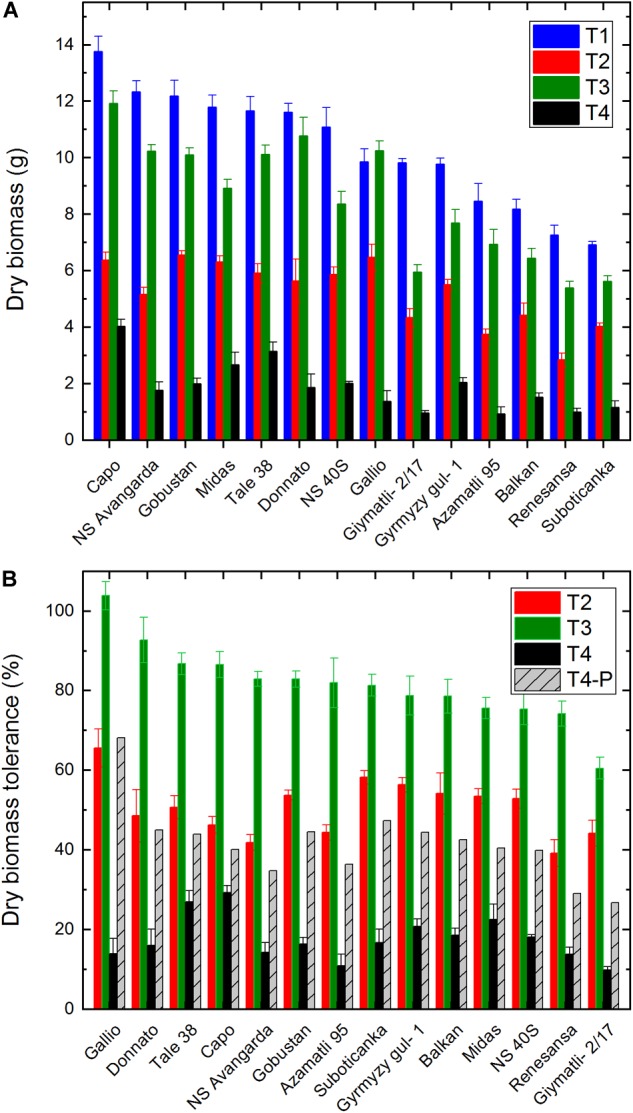
Effect of salt and drought stress on total biomass and biomass tolerance in wheat plants. Dry biomass of the same population of wheat plants, which were used for the digital phenotyping, was measured at the end of the cultivation period. **(A)** The total dry biomass of the above-ground parts of plants is shown for the well watered (T1), water limited (T2), well watered plus salt (T3), and water limited plus salt (T4) conditions. **(B)** Stress tolerance of dry biomass production for each cultivar was calculated as percentage of biomass obtained under water limited (T2), well watered plus salt (T3), and water limited plus salt (T4) conditions relative to the biomass of the same variety obtained under well watered conditions, as described in the Section “Materials and Methods” (Equation 1). The T4-P column shows the extent of biomass tolerance, which is expected if salinity and drought would exert their biomass retardation effects independently of each other. Data shown are means ± SE (*n* = 5 plants/treatment). Statistical analysis of data is presented in Supplementary Table 4.

Total aboveground dry biomass, which was determined at the end of the cultivation period showed an almost 100% variation between the highest (‘Capo’) and lowest (‘Suboticanka’) biomass producing cultivars (Figure 2A). Mild salinity alone had only a relatively small effect, ca. 18% loss in average, while drought induced in average a ca. 50% biomass loss. When drought and salinity was applied simultaneously the biomass loss was ca. 82% in average (Figure 2B). As regards relative tolerance of biomass, i.e., the percentage of retained biomass in the stressed plants as compared to the well watered control, ‘Gallio’ showed the best performance both in case of T2 and T3 treatments (Figure 2B). The highest biomass decrease by salt stress was induced for ‘Gíymatli-2/17’ (ca. 40%), while drought affected ‘NS Avangarda’ and ‘Renesansa’ to the largest extent (ca. 60% loss). ‘Capo’ and ‘Tale 38’ showed the best resistance to the combined T4 treatment (ca. 70% loss), while ‘Gíymatli-2/17’ and ‘Azamatli 95’ lost 90% of their biomass (Figure 2B). The T4-P columns show the predicted biomass tolerance, which was calculated on the assumption that salinity and drought induced biomass losses occurred independently from each other. It is very important to note that the actual tolerance of biomass under T4 condition is ca. twofold smaller, than that predicted by assuming non-interacting effects of salinity and drought.

In a recent work the combined effect of strong salinity (100 mM applied form the 25th day of the experiment) and strong water stress (water withdrawal leading to 4% relative soil humidity by the 45th day of the experiment) was studied in two wheat cultivars (Dugasa et al., 2018). Under these conditions only small enhancement, or even a small protection, was observed in the decrease in the plant height and shoot biomass in comparison to the individually applied salinity and drought stresses (Dugasa et al., 2018). These data indicate that the interacting effects of salinity and drought can be more pronounced in the presence of mild salinity and medium level drought as was shown in our study.

**FIGURE 3:**
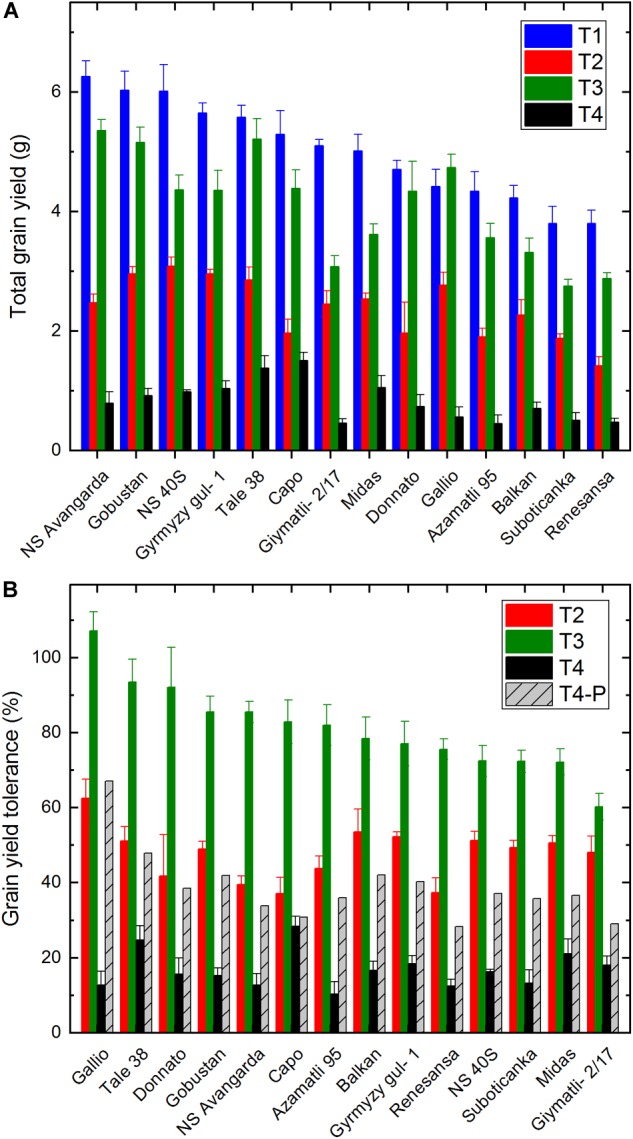
Effect of salt and drought stress on total grain yield and grain yield tolerance in wheat plants. Total grain yield of the same population of wheat plants, which were used for the digital phenotyping and biomass determination, was measured at the end of the cultivation period. **(A)** The total grain yield is shown for the well watered (T1), water limited (T2), well watered plus salt (T3), and water limited plus salt (T4) conditions. **(B)** Stress tolerance of grain yield for each cultivar, was calculated as percentage of grain yield obtained under water limited (T2), well watered plus salt (T3), and water limited plus salt (T4) conditions relative to the grain yield of the same variety obtained under well watered conditions, as described in the Section “Materials and Methods” (Equation 1). The T4-P column shows the extent of grain yield tolerance, which is expected if salinity and drought would exert their yield retardation effects independently of each other. Data shown are means ± SE (*n* = 5 plants/treatment). Statistical analysis of data is presented in Supplementary Table 5.

Grain production in case of wheat is more important than the overall above ground biomass, and the total grain yield could be considered as the most important yield parameter for agriculture. Therefore, at the end of the cultivation period the grain yield from the main spike, as well as the yield from the side tillers was also determined. In absolute values the ‘NS Avangarda,’ ‘NS 40S’ and ‘Gobustan’ cultivars showed the highest grain yield values under T1 control conditions, while the lowest yield was observed for ‘Suboticanka’ and ‘Renesansa’ (Figure 3A). Similarly to green leaf area and dry biomass the smallest yield loss was induced by salinity, T3 (in average ca. 18%). In absolute terms the highest grain yield under T3 was realized by ‘NS Avangarda’ and ‘Tale 38,’ while the lowest yields by ‘Suboticanka’ and ‘Renesansa.’ Under drought the best performing lines were ‘NS 40S,’ ‘Gobustan’ and ‘Gymyzy gul-1,’ while ‘Renesansa’ performed worst. The most drastic effect was observed under the combined T4 treatment (Figure 3A). Highest total grain yield in this case was observed in the ‘Capo’ and ‘Tale 38,’ the lowest for ‘Azamatli 95’ and ‘Renasansa’ cultivars (Figure 3A).

The relative loss of grain yield under stress conditions as compared to the well watered conditions is a measure of yield tolerance. As shown in Figure 3B the tolerance of grain yield varied between 106% (‘Gallio’) and 60% (‘Giymatli-2/17’) under saline (T3) conditions. Under drought, T2, ‘Gallio’ showed the highest (62%), while ‘Capo’ and ‘Renesansa’ the lowest (38%) relative tolerance of the grain yield. Considering those cultivars, which retain at least 50% of their control grain yield under the applied drought conditions being drought tolerant the ‘Gallio,’ ‘Balkan,’ ‘Gyrmyzy gul-1,’ ‘Midas,’ ‘Gobustan,’‘NS 40S,’ belongs to this category, this classification shows a good general agreement with the literature data (Table 1). However, in some cases, which have close to 50% grain yield tolerance (‘Tale 38,’ ‘Gyrmyzy gul-1’) the literature classification is not tolerant, while in case ‘Azamatli 95,’ which in our case retains somewhat less than 50% of the control grain yield, the literature classification is tolerant (Talai, 2010).

The most serious yield loss was induced by the combined T4 treatment. Under these conditions ‘Capo’ showed the best (39%) and ‘Azamatli 95’ the worst (11%) yield tolerance, while the average tolerance of all cultivars was 18%.

Similarly to that observed for biomass, the tolerance of grain yield under the T4 condition was only ca. half of that was predicted by assuming non-interacting effects of salinity and drought (T4-P). These data demonstrate that mild salinity and drought interact with each other and synergistically suppress biomass and grain yield, well below the level that could be expected from non-interacting effects. Interestingly, this interacting effect is largely cultivar dependent and quite small (factor of 1.1) in case of Capo, and very big (factor of 5.2) in case of Gallio (Figure 2B, 3B).

### Predictive Power of Leaf Area Monitoring for Estimation of Dry Biomass and Grain Yield

**FIGURE 4:**
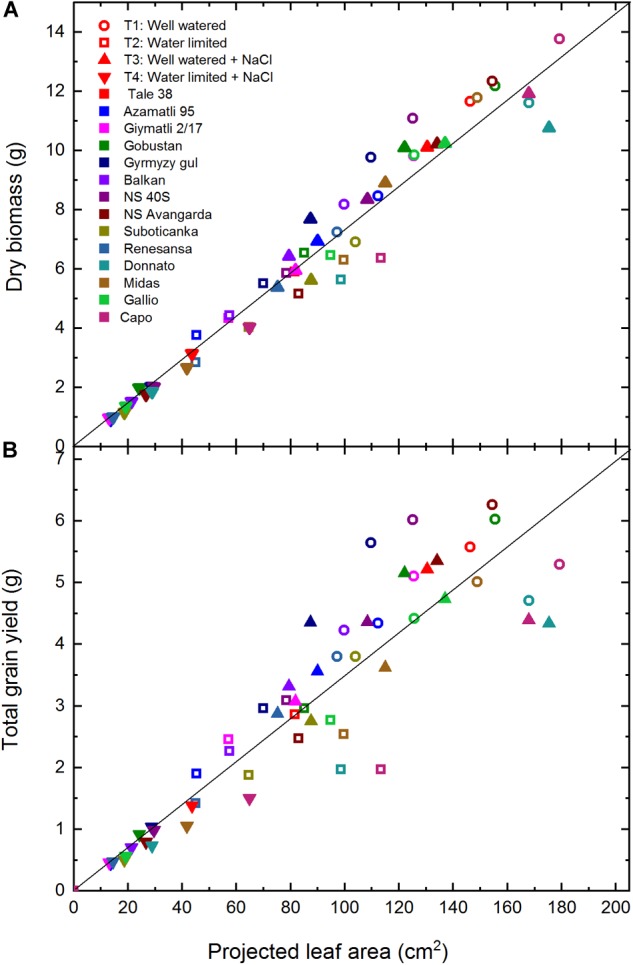
Correlation of projected leaf area with dry biomass and total grain yield. Total dry biomass **(A)** and total grain yield **(B)** are plotted as a function of projected leaf area for all wheat cultivars obtained under well watered (T1), water limited (T2), well watered plus salt (T3), and water limited plus salt (T4) conditions. The shape of the symbols corresponds to the treatments, while the color code represents the different cultivars. Data shown are mean of *n* = 5 plants/treatment. The solid lines represent the ideal linear correlation trendline. Statistical analysis (heteroscedasticity tests for the distribution of residuals) is presented in Supplementary Table 2.

The basic idea behind the application of various imaging approaches to follow shoot/leaf development is that they can be used for predictions of important agricultural traits, such as biomass, and grain yield at the end of the cultivation period. In order to check the predictive potential of the projected green leaf/shoot area we used the values obtained by taking the average of leaf area values obtained on three different days between the 30–45 days of the experiment, where all cultivars were close to or reached the maximal value of this parameters, and compared it with the total dry biomass and grain yield determined at the end of the experiment (75–80 days). According to Figure 4A, the dry biomass shows a reasonably good linear correlation with the projected leaf area for the complete data set, which includes all studied cultivars under the four experimental conditions. There are only few outliers from the general trend, e.g., ‘Capo’ and ‘Donnato,’ whose dry biomass falls below the general trend line. This difference most likely reflects the specific morphological characteristics of ‘Donnato’ and ‘Capo,’ i.e., relative large leaf area with thin tissue.

Correlation of total grain yield with projected leaf area (Figure 4B) is much less strict than seen for dry biomass (Figure 4A). The data obtained for ‘Capo,’ ‘Midas’ and ‘Donnato’ are well below the line corresponding to the general trend, which shows that in these cultivars a larger canopy is required to produce the same amount of grains compared to the other cultivars. Interestingly, the correlation of leaf area with either total biomass or grain yield is very strict under the severe conditions of simultaneous salt and drought stresses, and breaks down gradually when the plants are grown under less stressful conditions.

The above data show that non-destructive shoot/leaf imaging is a very useful approach to obtain data on above ground biomass of wheat. This method reflects well the biomass differences of different cultivars under the same growth conditions, and also under different stress conditions. On the other hand, the predictive potential of shoot/leaf imaging is much weaker when the target is the estimation of grain yield. Although an overall correlation exists between projected leaf area and grain yield this correlation is not sufficient to predict the large differences in grain yield belonging to cultivars with approximately the same leaf area (Figure 4B). Therefore, in phenotyping studies where the target is grain yield optimization conclusions based on projected leaf area should be made with caution in agreement with earlier findings (Paul et al., 2016).

### Effects of Salt and Drought Stress on Allocation of Photosynthates to Biomass and Grains

**FIGURE 5:**
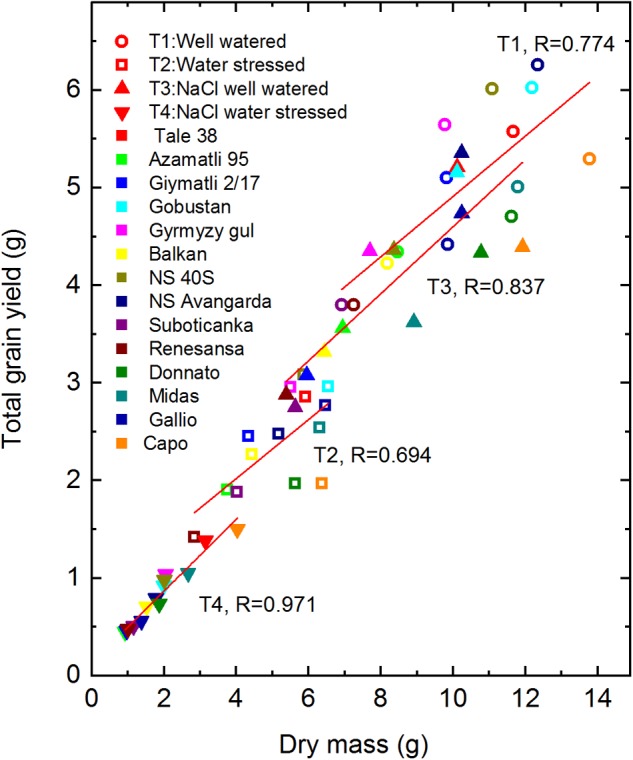
Correlation of dry biomass and total grain yield. Total grain yield is plotted as a function of total dry biomass projected for all wheat cultivars obtained under well watered (T1), water limited (T2), well watered plus salt (T3), and water limited plus salt (T4) conditions. The shape of the symbols corresponds to the treatments, while the color code represents the different cultivars. Data shown are means ± SE (*n* = 5 plants/treatment). The red solid lines represent the best fitting linear correlation curves for each of the four treatments with the indicated Pearson’s *R* values. Statistical analysis (heteroscedasticity tests for the distribution of residuals) is presented in Supplementary Table 2.

The data obtained for dry biomass and grain yield can also be used for the analysis of the drought- and salt stress induced effects on the efficiency of allocating photosynthates to overall biomass and to grain production. As shown in Figure 5, there is an overall trend for correlation between grain yield and dry biomass. However, the quality of the correlation depends on the applied stress conditions. Interestingly, almost perfect linear correlation (*R* = 0.971) was observed for the combined T4 treatment, which produced the lowest biomass and grain yield. In the T1 control conditions the tendency for linear correlation was maintained (*R* = 0.774), but variability in the grain yield belonging to roughly the same biomass was much larger (reaching 30%, in case ‘Donnato’ vs. ‘NS 40S’ and ‘Gobustan’). In case of T2 and T3 the correlation was also less strict (*R* = 0.837 and 0.694, respectively) than of T4. The variation of grain yield at the same total biomass reflects natural variation in the efficiency of partitioning biomass into the grains. The interesting observation is that under stress conditions, especially under the combined drought- and salt stress, this variation is largely suppressed, indicating that under stressful conditions plants can mobilize less of their extra resources for grain production than under optimal growth conditions.

**FIGURE 6:**
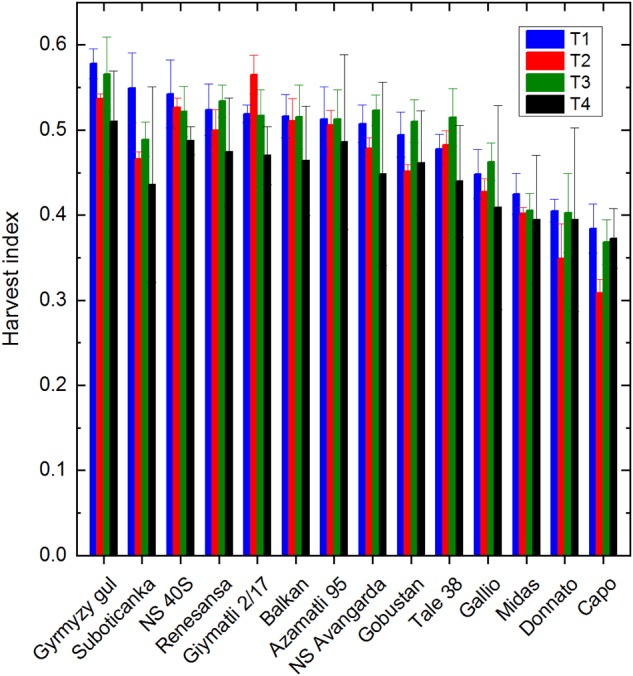
Effect of drought and salt stress on the Harvest index. Harvest index was calculated from the ratio of total grain yield and total dry biomass, and shown for the different treatments: T1 (well watered), T2 (water limited), T3 (salt, well watered), T4 (salt, water limited). Data shown are means ± SE (*n* = 5 plants/treatment). Statistical analysis of data is presented in Supplementary Table 6.

The harvest index (HI) values are ranging from 0.55–0.57 (‘Gymyzy gul,’ ‘Suboticanka’) to 0.38–0.40 (‘Capo,’ ‘Donnato’) in the T1 control (Figure 6). These values are decreased somewhat by (0.05–0.10) by drought in some cultivars (‘Suboticanka,’ ‘Donnato,’ ‘Capo’). Salinity decreased the HI value only in ‘Suboticanka’ when applied alone, but affected almost all cultivars when applied together with drought by (0.05–0.15). However, in some cultivars (‘Donnato,’ ‘Midas,’ ‘Capo’), the HI value was practically the same under T4 as in T1. Taken together, these data show that even under very severe stress, when the total dry biomass decreases to less than 10% of that produced under control conditions the plants keep up the relative efficiency of seed production in order to survive the harsh conditions. These data are in agreement with earlier results obtained under salinity (Harris et al., 2010).

### Correlation of Drought and Salt Tolerance

Both salt and drought stress have osmotic and oxidative components, therefore, it is an interesting question if drought and salt tolerance shows similar tendency in the different cultivars, or not? Plotting the total dry biomass obtained under T3 and T4 treatments as a function of dry biomass obtained under drought, T2, shows a reasonably good positive correlation tendency (Supplementary Figure 2A). Similar result is obtained for the total grain yield (Supplementary Figure 2B). The situation is different when the stress tolerance of the yield parameters is concerned. In case of both total biomass and grain yield there is weaker correlation between the extent of stress tolerance of the yield parameters observed under drought and saline conditions, which practically disappears under simultaneous salt and drought stress (Supplementary Figures 3A,B).

These data show that those cultivars, which have high total biomass and grain yield under drought are expected to have high total biomass and grain yield also under saline, and saline + drought conditions. However, the relative tolerance of total biomass and grain yield observed under drought has only very weak prediction potential for biomass and grain yield expected under salt, and salt plus drought conditions. In the literature there are several examples showing that specific genes and compounds can protect against the consequences of both salinity and drought stress. These include MYB transcription factors (Rahaie et al., 2010; Qin et al., 2012; Wei et al., 2017; Du et al., 2018), sucrose non-fermenting1-related protein kinase 2 (SnRK2) (Zhang et al., 2010), *Triticum aestivum* salt-induced protein (TaSIP) (Du et al., 2013), a NAC transcription factor (TaNAC29) (Huang et al., 2015), dehidrins (Qin and Qin, 2016), the osmoprotectant D-pinitol (Ahn et al., 2018), an Auxin Response Factor Gene (IbARF5) (Kang et al., 2018), etc. The involved action mechanisms typically include antioxidative defense (MYB TFs, IbARF5), osmoprotection (MYB TFs, SnRK2, D-pinitol), ABA-dependent signaling (dehydrins) and also so far unknown mechanisms (TaSIP) and can explain the similar tendency for biomass and grain production under salt and drought stress. Salinity besides inducing osmotic effect also acts via ion toxicity (Munns and James, 2003; Munns and Tester, 2008; Arzani and Ashraf, 2016). Although the ionic effect is expected to be small under mildly saline conditions it might contribute to the retardation of biomass and grain explaining the weaker correlation between the relative tolerance against salinity and drought. In case of co-occurring salinity and drought the situation might be complicated even further by the exhaustion of antioxidant capacity in the simultaneous presence of the two stress factors as discussed below, which breaks further down the correlation of yield tolerance with that observed under drought alone.

### Effects of Salt and Drought Stress on Water Usage

**FIGURE 7:**
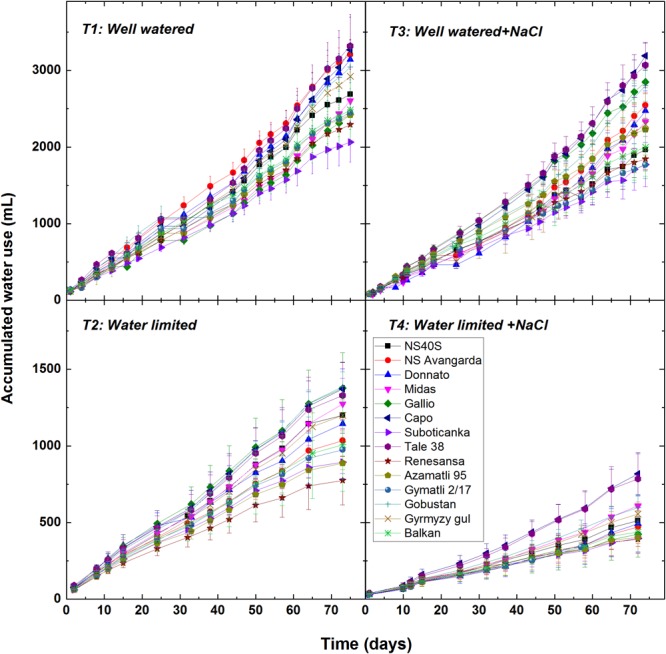
Effect of salt and drought stress on the water use of wheat plants. Computer controlled watering was used to determine the water use of individual plants of the selected 14 cultivars under well watered (T1), water limited (T2), salt plus well watered (T3), and salt plus water limited (T4) conditions. Data shown are means ± SE (*n* = 5 plants/treatment). Statistical analysis of data for the final point of the experiment is presented in Supplementary Table 7.

An important feature of the complex phenotyping approach used in our system is the precise water management at the level of individual plants. This was achieved by computer controlled watering of each plant to maintain the preset soil water capacity (20 and 60% for water limited and well watered conditions, respectively). This approach made possible to record the accumulated water usage for each plant, most of which was lost through transpiration. The amount of transpired water was quite different among the cultivars (Figure 7) showing up the 75% difference between the low (‘Suboticanka’) and high water consuming cultivars (‘Capo,’ ‘Tale 38’). Similarly to the other phenotypic markers water usage was only slightly affected by the T3 salt treatment, and was 80% that of the T1 control. Drought stress decreased the water usage by ca. 50%, while under the combined T4 treatment plants used less than 20% of water as compared to the T1 control.

**FIGURE 8:**
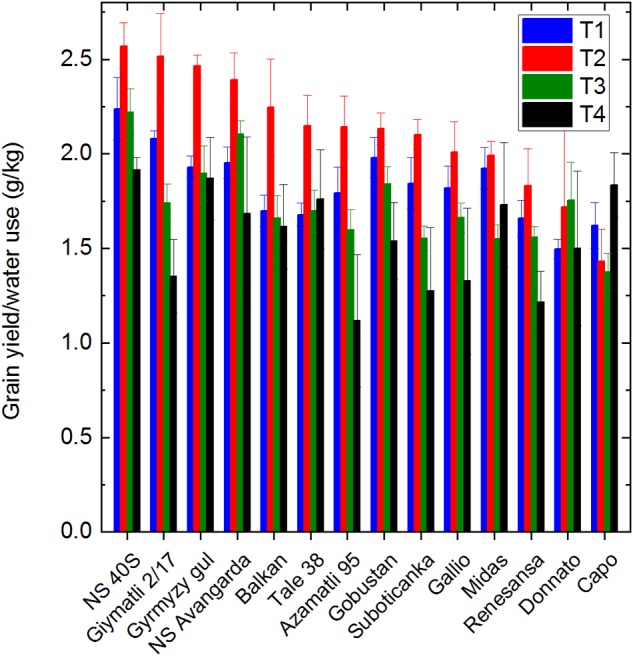
Effect of salt and drought stress on the efficiency water utilization for grain production. The ratio of total grain yield and the amount of water used during the whole cultivation period is shown for the selected 14 cultivars under well watered (T1), water limited (T2), salt plus well watered (T3), and salt plus water limited (T4) conditions. Data shown are means ± SE (*n* = 5 plants/treatment).

The efficiency of plants to utilize water for grain production can be estimated by the ratio of grain yield and of the used water. The data in Figure 8 show that in average 1.8 g grains were produced by transpiring 1 kg (1L) water under the T1 control conditions. This value was increased to ca. 2.1 g/kg under T2, and was decreased slightly (1.7 g/kg), or more seriously (1.5 g/kg) under T3 or T4 treatment, respectively. The ‘NS 40S’ and ‘NS Avangarda’ cultivars showed the highest efficiency in using water for grain production, while ‘Renesansa’ and ‘Capo’ showed the lowest values. These data show that the transpiration activity of wheat plants was affected to a smaller extent by the co-occurring salinity and drought than their grain producing efficiency.

### Effect of Drought and Salt Stress on Photosynthetic Activity

**FIGURE 9:**
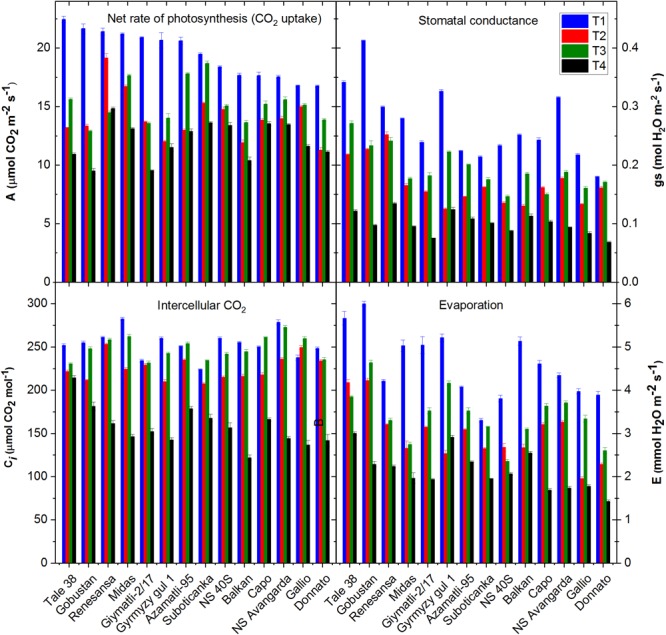
Effect of salt and drought stress on the gas exchange parameters. A Licor 6400 gas analyzer was used to determine the rate of CO_2_ uptake, stomatal conductance, intercellular CO_2_ concentration and the rate of evaporative water loss. The data obtained for the 14 selected wheat cultivars is shown under well watered (T1), water limited (T2), salt plus well watered (T3), and salt plus water limited (T4) conditions. Data shown are means ± SE (*n* = 10–12 repetitions on 5 different plants). Statistical analysis of data is presented in Supplementary Tables 8–11.

The basic process of biomass and grain production is photosynthetic light energy conversion. The efficiency of this process was quantified by measuring the rate of net photosynthesis via CO_2_ fixation under the applied stress conditions. Similarly to that observed in case of projected leaf/shoot and grain yield the combined T4 treatment caused the largest loss of net photosynthesis as compared to mild salinity (T3) and drought (T2) alone (Figure 9). However, the effect of T4 treatment relative to the T1 control and to the single T2 and T3 treatments was not so pronounced as seen for the leaf area and grain yield. In case of ‘Capo,’ ‘Suboticanka’ and ‘NS Avangarda’ the net CO_2_ uptake rate was maintained at 75–80% level of the well watered control and it decreased to 45–50% in case of ‘Glymatli 2/17’ and ‘Gobustan.’ The effects of salinity and drought on photosynthesis are attributed among other factors to the stomatal limitations for diffusion of gasses, which ultimately alters photosynthesis and the mesophyll metabolism (Parida et al., 2005; Chaves et al., 2009). Under mild stress, a small decline in stomatal conductance may have protective effects against stress, by allowing plant water saving and improving plant water-use efficiency by the plant (Chaves et al., 2009). However, under conditions of prolonged exposure to salt and/or drought stress stomatal limitation decreases the net rate of photosynthesis, as well as biomass and grain yield, which was indeed very clearly seen in our data. In almost all cultivars in our study drought induced larger loss of stomatal conductance than mild salinity (Figure 9). The simultaneous T4 treatment induced a large increase of stomatal closure in most of the studied cultivars, but in some cases (‘Gyrmyzy gul 1,’ ‘Balkan’) the effect of the double stress was practically the same as of drought alone. Plotting the net rate of photosynthesis as a function of stomatal conductance shows an approximate linear correlation between the two parameters (Supplementary Figure 4), which confirms that the primary cause for the decrease of net photosynthesis rate is the closure of stomata.

The intercellular CO_2_ concentration calculated from the CO_2_ gas exchange data is also an important parameter since it provides information on the site where limitation of net photosynthetic activity occurs under the applied stress conditions. Decrease of the intercellular CO_2_ concentration under stress exposure indicates that photosynthesis is limited more by the decrease of stomatal conductance than by the decrease of biochemical activity of the Calvin-Benson cycle. The opposite case, i.e., an increase of the intercellular CO_2_ level indicates a primary effect at the level of the biochemical activity, since CO_2_ can still enter the leaf tissue via the stomata, but cannot be utilized by the CO_2_ fixing biochemical processes (Lauer and Boyer, 1992). The data show that drought alone and especially in combination with salinity decreases the intercellular CO_2_ level in all wheat cultivars (Figure 9), confirming that the main cause of decreased net photosynthetic activity under T2 and T4 treatments is the closure of stomata, which is in agreement with the general correlation of the rate of net photosynthesis and stomatal conductance (Supplementary Figure 4). Under saline, T3, conditions the decrease of the intercellular CO_2_ concentration is much less than observed for the combined T4 treatment, which is in agreement with the smaller effect on stomatal conductance.

Photosystem II (PSII) is the final source of electrons for photosynthesis and its function is also prone to damage by various stress factors. The rate of electron transport through PSII is indicative of PSII activity itself and also of its limitation by electron sinks, especially the Calvin-Benson cycle. The light intensity dependence of ETR(II) is shown for two cultivars in Supplementary Figure 5. As seen for other parameters, ETR(II) is decreased most by the combined T4 treatment. Under these conditions saturation occurred at lower light intensities and at a lower saturation levels than in the T1 control or in the T2 and T3 treatments. These data are consistent with the limited terminal electron sinks, most likely at the level of the Calvin-Benson cycle, under the salt plus drought stress conditions.

### Proline Accumulation

**FIGURE 10:**
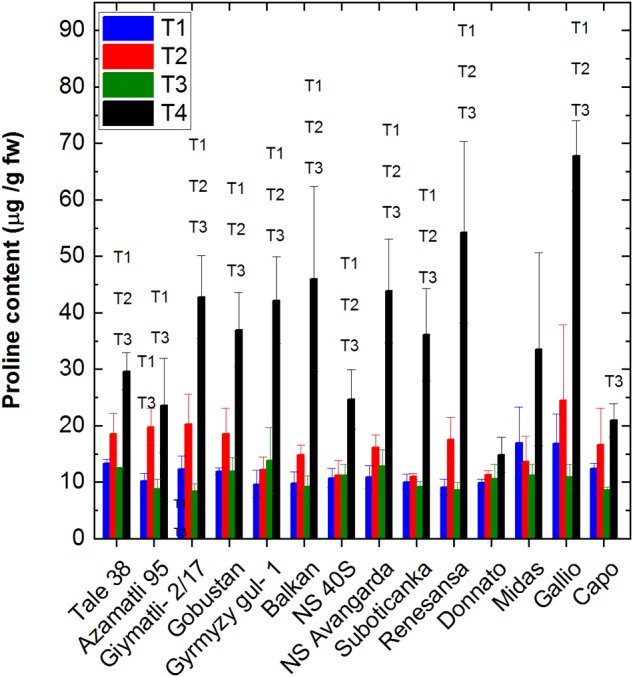
Effect of drought and salt stress on proline accumulation. Proline content was determined from leaf samples collected the fully developed leaves below the flag leaves at the 10th week after the start of the stress treatments. Free proline content was determined according to Bates et al. (1973) for the 14 selected wheat cultivars under well watered (T1), water limited (T2), salt plus well watered (T3), and salt plus water limited (T4) conditions. Data shown are means ± SE (*n* = 5 plants/treatment). Statistical significance of T4 with respect to other treatment conditions T1, T2, and T3 were indicated. Statistical analysis of data is presented in Supplementary Table 12.

Proline accumulation is one of the common characteristics in many monocotyledons under water deficit, salinity and oxidative stress conditions (Szabados and Savouré, 2010). An increase of proline content was detected under drought stress in our study, especially in genotypes of ‘Tale 38,’ ‘Azamatli 95,’ ‘Giymatli 2/17,’ and ‘Gallio,’ but the effect was statistically significant only for ‘Giymatli 2/17.’ Increase of proline under T4 in comparison to T1 was significant in all cultivars with the exception of ‘Midas,’ and showed the largest effect in ‘Gallio’ (Figure 10 and Supplementary Table 11). It is assumed that the accumulation of proline is an adaptation response partly to provide osmoprotection as well as to decrease the damaging effects caused by reactive oxygen species, which are produced due to limitation of photosynthesis induced by stomatal closure (Szabados and Savouré, 2010). Since the mild level of salinity used in our experiments did not induce significant proline accumulation when applied alone we can conclude that accumulation of salt ions was negligible under our mild saline conditions. Also, drought alone induced only a statistically insignificant increase of proline (Figure 10). Therefore, the proline data indicate an enhanced osmotic and/or oxidative effect only under the combined application of salt and drought stress, which is not compensated by other defense mechanisms.

It is also interesting to observe that proline induction was relatively small in cultivars, such as ‘Capo’ and ‘Tale 38,’ which showed the highest tolerance of biomass and grain yield under the combined T4 treatment. This indicates that these cultivars were effectively preventing the formation of harmful oxidative conditions by other antioxidant systems without the need for a large extent of proline production. In contrast, cultivars, such as ‘Gallio,’ which suffered large biomass and grain yield loss under the double stress conditions showed very high level of proline production, indicating the inefficiency of other antioxidant protective mechanisms. Limitation of antioxidant response might also explain at least partly the large synergistic retardation of biomass and grain production, i.e., difference between the measured and predicted yield loss values under the T4 treatment, observed in ‘Gallio’ (Figure 2B, 3B). Exhaustion of the antioxidative defense capacity by the co-occurring salinity and drought can lead to a situation of largely enhanced damage in ‘Gallio.’ In the opposite case of ‘Capo’ and ‘Tale 38’ the antioxidative defense capacity seems to be sufficient to combat not only the effects of salinity and drought when occur separately, but also when they occur together. Therefore, the yield loss under the combined stress conditions is only slightly exceeds the level, which is expected by assuming non-interacting damage mechanism by salinity and drought.

## Conclusion

In our study we used a greenhouse based phenotyping platform to obtain detailed data about the salt and drought stress induced responses of 14 wheat cultivars originating from different geographical locations, regarding their green and dry biomass, grain yield, water consumption, photosynthetic activity, and proline accumulation.

Our data show that RGB imaging, which is the most commonly used phenotyping approach, provides very useful data for the estimation of biomass accumulation even if applied only in part of the plant development period. On the other hand RGB imaging of leaf/shoot area has only a limited predictive potential for grain yield. Therefore, the actual determination of grain yield cannot be replaced with shoot/leaf imaging when grain yield optimization is the target of the project.

The presence of a mild level salinity, 2 g NaCl/kg soil, induced only a small effect (ca. 18% loss in average) in the phenotypic parameters under well watered conditions. A larger adverse effect was induced by drought stress (50% loss in average). Importantly, a large enhancement of biomass and grain yield loss (83% in average) was observed when salinity was combined with drought stress. The actual yield loss was twofold larger in average than that would be expected if salinity and drought were exerting their effects independently. These data show for the first time that salinity and drought can have synergistically interacting adverse effects on biomass and grain yield. The mechanism of this interaction is not clear at present, but cultivar-dependent differences, which are correlated with proline accumulation, indicate that it might be related to limitations of antioxidative defense capacity. Future studies at the molecular level, including Na^+^ and Cl^-^ determination in the leaves, are required to clarify the actual mechanism behind the interacting effects of salinity and drought.

The finding of interacting effects of mild salinity and drought have important consequences for agriculture since it shows that when mildly saline areas are affected by drought the crop yield loss can be aggravated. However, the large differences among the cultivars shown by our data in the level of their tolerance against the combination of salt and drought stress indicates an important potential for identifying the involved defense mechanisms and also for selection or creation of wheat cultivars, which show higher tolerance against these conditions.

## Author Contributions

All authors prepared, read, and approved the final manuscript. IV, JP, AK-S, and HG proposed the research topic, designed the experiments, and contributed to writing of the manuscript. KP conducted most of the experiments, prepared the figures, and wrote the manuscript draft. LS and TA performed part of the experiments and reviewed drafts of the manuscript.

## Conflict of Interest Statement

The authors declare that the research was conducted in the absence of any commercial or financial relationships that could be construed as a potential conflict of interest.

## Footnotes

1 https://www.xlstat.com

## References

[B1] Addinsoft (2019). *XLSTAT.* Available at: https://www.xlstat.com

[B2] Ahn C. H. Hossain M. A. Lee E. Kanth B. K. Park P. B. (2018). Increased salt and drought tolerance by D-pinitol production in transgenic *Arabidopsis thaliana*. *Biochem. Biophys. Res. Commun.* 504 315–320. 10.1016/j.bbrc.2018.08.183 30180952

[B3] Al-Tamini N. Brien C. Oakey H. Berger B. Saade S. Ho Y. S. (2016). Salinity tolerance loci revealed in rice using high-throughput non-invasive phenotyping. *Nat. Commun* 7:13342. 10.1038/ncomms13342 27853175 PMC5118543

[B4] Arzani A. Ashraf M. (2016). Smart engineering of genetic resources for enhanced salinity tolerance in crop plants. *Crit. Rev. Plant Sci.* 35 146–189. 10.1080/07352689.2016.1245056

[B5] Ashraf M. Harris P. J. C. (2013). Photosynthesis under stressful environments: an overview. *Photosynthetica* 51 163–190. 10.1111/plb.12014 23574304

[B6] Babayev H. G. Bayramov S. M. Mehvaliyeva U. A. Aliyeva M. N. Guliyev N. M. Huseynova I. M. (2013). Activities of C4-photosynthetic enzymes in different wheat genotypes under continuous soil drought conditions. *J. Biochem. Res* 1 7–16.

[B7] Babic L. Babic M. Turan J. Matic-Kekic S. Radojcin M. Mehandzic-Stanisic S. (2011). Physical and stress-strain properties of wheat (*Triticum aestivum*) kernel. *J. Sci. Food Agric.* 91 1236–1243. 10.1002/jsfa.4305 21328363

[B8] Bates L. S. Waldren R. P. Teare I. D. (1973). Rapid determination of free proline for water-stress studies. *Plant Soil* 39 205–207. 10.1016/j.dental.2010.07.006 20688380 PMC2948575

[B9] Björkman O. Demmig B. (1987). Photon yield of O2 evolution and chlorophyll fluorescence characteristics at 77 K among vascular plants of diverse origins. *Planta* 170 489–504. 10.1007/BF00402983 24233012

[B10] Chaves M. M. Flexas J. Pincheiro C. (2009). Photosynthesis under drought and salt stress: regulation mechanisms from whole plant to cell. *Ann. Bot.* 103 551–560. 10.1093/aob/mcn125 18662937 PMC2707345

[B11] Chen C. S. Xie Z. X. Liu X. J. (2009). Interactive effects of drought and salt stresses on winter wheat seedlings growth and physiological characteristics of stress-resistance. *J. Appl. Ecol.* 20 811–816. 19565760

[B12] Cobb J. N. DeClerck G. Greenberg A. Clark R. McCouch S. (2013). Next-generation phenotyping: requirements and strategies for enhancing our understanding of genotype-phenotype relationships and its relevance to crop improvement. *Theor. Appl. Genet.* 126 867–887. 10.1007/s00122-013-2066-0 23471459 PMC3607725

[B13] Cseri A. Sass L. Törjék O. Pauk J. Vass I. Dudits D. (2013). Monitoring drought responses of barley genotypes with semi-robotic phenotyping platform and association analysis between recorded traits and allelic variants of some stress genes. *Aust. J. Crop Sci.* 7 1560–1570.

[B14] Dencic S. Kastori R. Kobiljski B. Duggan B. (2000). Evaluation of grain yield and its components in wheat cultivars and landraces under near optimal and drought conditions. *Euphytica* 113 43–52. 10.1023/A:1003997700865

[B15] Dimitrijevic M. Petrovic S. Mladenov N. Belic M. Hristov N. Banjac B. (2009). Phenotyping reaction of wheat grown on different soil types. *Genetika* 41 169–177. 10.2298/GENSR0902169D

[B16] Du H. Y. Shen Y. Z. Huang Z. J. (2013). Function of the wheat TaSIP gene in enhancing drought and salt tolerance in transgenic *Arabidopsis* and rice. *Plant Mol. Biol.* 81 417–429. 10.1007/s11103-013-0011-x 23400831

[B17] Du Y. T. Zhao M. J. Wang C. T. Gao Y. Wang Y. X. Liu Y. W. (2018). Identification and characterization of GmMYB118 responses to drought and salt stress. *BMC Plant Biol.* 18:320. 10.1186/s12870-018-1551-7 30509166 PMC6276260

[B18] Dugasa M. T. Cao F. Ibrahim W. Wu F. (2018). Differences in physiological and biochemical characteristics in response to single and combined drought and salinity stresses between wheat genotypes differing in salt tolerance. *Physiol. Plant* 165 134–143. 10.1111/ppl.12743 29635753

[B19] Fehér-Juhász E. Majer P. Sass L. Lantos C. Csiszár J. Turóczy Z. (2014). Phenotyping shows improved physiological traits and seed yield of transgenic wheat plants expressing the alfalfa aldose reductase under permanent drought stress. *Acta Physiol. Plant* 36 663–673. 10.1007/s11738-013-1445-0

[B20] Flexas J. Bota J. Loreto F. Cornic G. Sharkey T. D. (2004). Diffusive and metabolic limitations to photosynthesis under drought and salinity in C3 plants. *Plant Biol.* 6 1–11. 10.1055/s-2004-820867 15143435

[B21] Flexas J. Diaz-Espejo A. Galmes J. Kaldenhoff R. Medrano H. Ribas-Carbo M. (2007). Rapid variations of mesophyll conductance in response to changes in CO2 concentration around leaves. *Plant Cell Environ.* 30 1284–1298. 10.1111/j.1365-3040.2007.01700.x 17727418

[B22] Flowers T. J. Yeo A. R. (1995). Breeding for salinity resistance in crop plants: where next? *Aust. J. Plant Physiol.* 22 875–884. 10.1071/PP9950875

[B23] Furbank R. T. Tester M. (2011). Phenomics - technologies to relieve the phenotyping bottleneck. *Trends Plant Sci.* 16 635–644. 10.1016/j.tplants.2011.09.005 22074787

[B24] Genty B. Briantais J.-M. Baker N. R. (1989). The relationship between the quantum yield of photosynthetic electron transport and quenching of chlorophyll fluorescence. *Biochim. Biophys. Acta* 990 87–92. 10.1016/S0304-4165(89)80016-9

[B25] Ghanem M. E. Marrou H. Sinclair T. R. (2015). Physiological phenotyping of plants for crop improvement. *Trends Plant Sci.* 20 139–144. 10.1016/j.tplants.2014.11.006 25524213

[B26] Guan X.-K. Song L. Wang T.-C. Turner N. C. Li F.-M. (2015). Effect of drought on the gas exchange, chlorophyll fluorescence and yield of six different-era spring wheat cultivars. *J. Agro. Crop Sci.* 201 253–266. 10.1111/jac.12103

[B27] Harris B. N. Sadras V. O. Tester M. (2010). A water-centred framework to assess the effects of salinity on the growth and yield of wheat and barley. *Plant Soil* 336 377–389. 10.1007/s11104-010-0489-9

[B28] Huang Q. Wang Y. Li B. Chang J. Chen M. Li K. (2015). TaNAC29, a NAC transcription factor from wheat, enhances salt and drought tolerance in transgenic *Arabidopsis*. *BMC Plant Biol.* 15:268. 10.1186/s12870-015-0644-9 26536863 PMC4632686

[B29] Huseynova I. M. Suleymanov S. Y. Aliyev J. A. (2007). Structural-functional state of thylakoid membranes of wheat genotypes under water stress. *Biochim. Biophys. Acta* 1767 869–875. 10.1016/j.bbabio.2007.01.014 17321491

[B30] Kacira M. Ling P. P. (2001). Design and development of an automated and non-contact sensing system for continuous monitoring of plant health and growth. *Trans. ASAE* 44 989–996. 10.13031/2013.6231 12026934

[B31] Kang C. He S. Zhai H. Li R. Zhao N. Liu Q. (2018). A Sweetpotato auxin response factor gene (IbARF5) is involved in carotenoid biosynthesis and salt and drought tolerance in transgenic *Arabidopsis*. *Front. Plant Sci.* 9:1307. 10.3389/fpls.2018.01307 30254657 PMC6141746

[B32] Kumar D. Al Hassan M. Naranjo M. A. Agrawal V. Boscaiu M. Vicente O. (2017). Effects of salinity and drought on growth, ionic relations, compatible solutes and activation of antioxidant systems in oleander (*Nerium oleander* L.). *Plos One* 12:22. 10.1371/journal.pone.0185017 28922384 PMC5602669

[B33] Landi S. Hausman J. F. Guerriero G. Esposito S. (2017). Poaceae vs. abiotic stress: focus on drought and salt stress, recent insights and perspectives. *Front. Plant Sci.* 8:1214. 10.3389/fpls.2017.01214 28744298 PMC5504180

[B34] Lauer M. J. Boyer J. S. (1992). Internal CO2 measured directly in leaves. absisic acid and low leaf water pontential cause opposing effects. *Plant Physiol.* 98 1310–1316. 10.1104/pp.98.4.1310 16668793 PMC1080350

[B35] Lobell D. B. Schlenker W. Costa-Roberts J. (2011). Climate trends and global crop production since 1980. *Science* 333 616–620. 10.1126/science.1204531 21551030

[B36] Majer P. Sass L. Lelley T. Cseuz L. Vass I. Dudits D. (2008). Testing drought tolerance of wheat by a complex stress diagnostic system installed in greenhouse. *Acta Biol. Szegediensis* 52 97–100.

[B37] Maser P. Eckelman B. Vaidyanathan R. Horie T. Fairbairn D. J. Kubo M. (2002). Altered shoot/root Naţ distribution and bifurcating salt sensitivity in *Arabidopsis* by genetic disruption of the Naţ transporter AtHKT1. *FEBS Lett.* 531 157–161. 10.1016/S0014-5793(02)03488-9 12417304

[B38] Mittler R. (2006). Abiotic stress, the field environment and stress combination. *Trends Plant Sci.* 11 15–19. 10.1016/j.tplants.2005.11.002 16359910

[B39] Munns R. (2002). Comparative physiology of salt and water stress. *Plant Cell Environ.* 25 239–250. 10.1046/j.0016-8025.2001.00808.x 11841667

[B40] Munns R. James R. A. (2003). Screening methods for salinity tolerance: a case study with tetraploid wheat. *Plant Soil* 253 201–218. 10.1023/A:1024553303144

[B41] Munns R. Tester M. (2008). Mechanisms of salinity tolerance. *Annu. Rev. Plant Biol.* 59 651–681. 10.1146/annurev.arplant.59.032607.092911 18444910

[B42] Nevo E. Chen G. X. (2010). Drought and salt tolerances in wild relatives for wheat and barley improvement. *Plant Cell Environ.* 33 670–685. 10.1111/j.1365-3040.2009.02107.x 20040064

[B43] Parida A. K. Mittra B. Das A. B. Das T. K. Mohanty P. (2005). High salinity reduces the content of a highly abundant 23-kDa protein of the mangrove *Bruguiera parviflora*. *Planta* 221 135–140. 10.1007/s00425-004-1415-2 15580524

[B44] Paul K. Pauk J. Deák Z. Sass L. Vass I. (2016). Contrasting response of biomass and grain yield to severe drought in cappelle desprez and plainsman V wheat cultivars. *PeerJ* 4:e1708. 10.7717/peerj.1708 27047703 PMC4815492

[B45] Qin Y. Wang M. Tian Y. He W. Han L. Xia G. (2012). Over-expression of TaMYB33 encoding a novel wheat MYB transcription factor increases salt and drought tolerance in *Arabidopsis*. *Mol. Biol. Rep.* 39 7183–7192. 10.1007/s11033-012-1550-y 22350156

[B46] Qin Y. X. Qin F. (2016). Dehydrins from wheat x *Thinopyrum ponticum* amphiploid increase salinity and drought tolerance under their own inducible promoters without growth retardation. *Plant Physiol. Biochem.* 99 142–149. 10.1016/j.plaphy.2015.12.011 26756791

[B47] Rahaie M. Xue G. P. Naghavi M. R. Alizadeh H. Schenk P. M. (2010). A MYB gene from wheat (*Triticum aestivum* L.) is up-regulated during salt and drought stresses and differentially regulated between salt-tolerant and sensitive genotypes. *Plant Cell Rep.* 29 835–844. 10.1007/s00299-010-0868-y 20490502

[B48] Rajendram K. Tester M. Roy S. J. (2009). Quantifying the three main components of salinity tolerance in cereals. *Plant Cell Environ.* 32 237–249. 10.1111/j.1365-3040.2008.01916.x 19054352

[B49] Rengasamy P. North S. Smith A. (2010). *Diagnosis and Management of Sodicity and Salinity in Soil and Water in the Murray Irrigation Region.* Adelaide, SA: The University of Adelaide.

[B50] Roy S. T. Negrao S. Tester M. (2014). Salt resistant crop plants. *Curr. Opin. Biotech.* 26 115–124. 10.1016/j.copbio.2013.12.004 24679267

[B51] Salekdeh G. H. Reynolds M. Bennett J. Boyer J. (2009). Conceptual framework for drought phenotyping during molecular breeding. *Trends Plant Sci.* 14 488–496. 10.1016/j.tplants.2009.07.007 19716744

[B52] Schreiber U. (2004). *Pulse-Amplitude-Modulation (PAM) Fluorometry and Saturation Pulse method: an Overview, Chlorophyll a Fluorescence.* Berlin: Springer 279–319. 10.1007/978-1-4020-3218-9_11

[B53] Szabados L. Savouré A. (2010). Proline: a multifunctional amino acid. *Trends Plant Sci.* 15 89–97. 10.1016/j.tplants.2009.11.009 20036181

[B54] Talai J. M. (2010). Biological and economic peculiarities of newly developed wheat varieties. *Proc. ANAS* 65 216–223.

[B55] Teizer B. (2010). *Novel Selection Criteria For Drought Tolerant Winter Wheat Genotypes and Their Correlations to Drought Stress Indicators, Crop Development, Plant Morphology, Yield, and Quality Parameters.* Ph. D Thesis, Institute of Agronomy and Plant Breeding (IPP). University of Natural Resources and Life Sciences Vienna 1–95.

[B56] Tester M. Langridge P. (2010). Breeding technologies to increase crop production in a changing world. *Science* 327 818–822. 10.1126/science.1183700 20150489

[B57] Wang W. Vinocur B. Altman A. (2003). Plant responses to drought, salinity and extreme temperatures: towards genetic engineering for stress tolerance. *Planta* 218 1–14. 10.1007/s00425-003-1105-5 14513379

[B58] Wei Q. Luo Q. Wang R. Zhang F. He Y. Zhang Y. (2017). A wheat R2R3-type MYB transcription factor TaODORANT1 positively regulates drought and salt stress responses in transgenic tobacco plants. *Front. Plant Sci.* 8:1374. 10.3389/fpls.2017.01374 28848578 PMC5550715

[B59] Zhang H. Mao X. Wang C. Jing R. (2010). Overexpression of a common wheat gene TaSnRK2.8 enhances tolerance to drought, salt and low temperature in *Arabidopsis*. *PLoS One* 5:e16041. 10.1371/journal.pone.0016041 21209856 PMC3012728

[B60] Zhang X. K. Lu G. Y. Long W. H. Zou X. L. Li F. Nishio T. (2014). Recent progress in drought and salt tolerance studies in *Brassica* crops. *Breed. Sci.* 64 60–73. 10.1270/jsbbs.64.60 24987291 PMC4031111

