# Supplementary material for: Co-occurrence of Mild Salinity and Drought Synergistically Enhances Biomass and Grain Retardation in Wheat
Source: Front Plant Sci. 2019 Apr 24;10:501. doi: 10.3389/fpls.2019.00501 (PMC6503295; doi:10.3389/fpls.2019.00501)
Supplement: Supplementary file 1 [file Data_Sheet_1.pdf]

Supplementary Material

Co-occurrence of mild salinity and drought synergistically enhances biomass and grain retardation in wheat

Kenny Paul[1,#], János Pauk[2], Ankica Kondic-Spika[3], Heinrich Grausgruber[4], Tofig Allahverdiyev[5], László Sass[1] and Imre Vass[1]*

[1]Institute of Plant Biology, Biological Research Centre, Hungarian Academy of Sciences.
[2]Department of Biotechnology, Cereal Research Non-Profit Ltd., Szeged, Hungary.
[3]Institute of Field and Vegetable Crops, Novi Sad, Serbia.
[4]Department of Crop Sciences, University of Natural Resources and Life Sciences, Vienna, Austria.
[5]Research Institute of Crop Husbandry, Ministry of Agriculture of Azerbaijan Republic
[#]Current address: Reliance Research and Development Centre, Relience Corporate Park, Navi Mumbai, India
*Corresponding author: Imre Vass, Institute of Plant Biology, Biological Research Centre, Hungarian Academy of Sciences, H-6726 Szeged, Temesvári krt. 62, Hungary
Email: vass.imre@brc.mta.hu. Phone: +36-62-599-700

Experimental details
The experiments were conducted with 14 wheat (*Triticum aestivum* L.) cultivars from Serbia (5), Austria (4) and Azerbaijan (5), which were grown under four different water/salt treatment (T) conditions:
T1- Well watered (60 % soil water capacity) and no salt (NaCl) added,
T2- Water limited (20 % soil water capacity) and no salt (NaCl) added,
T3- Well watered (60 % soil water capacity) and saline conditions (0.2% NaCl, i.e. 2g /kg soil),
T4- Water limited (20 % soil water capacity) and saline conditions (0.2% NaCl, i.e. 2g /kg soil).

At the end of the experiment (13 weeks after the stress treatments were started) biomass and grain production parameters (total above-ground mass, grain yield) were determined.

Gas exchange parameters: CO_2 uptake rate, transpiration, stomatal conductance and intercellular CO_2 concentration were measured by using a Licor 6400 gas analyzer (Licor, USA). Two to three selected pieces of attached leaves from plant replicates under respective treatments were inserted into the gas cuvette for individual measurements (Paul et al., 2016).The gas cuvette conditions were set to 400 ppm CO_2, ambient temperature and growth light intensity of photosynthetic active radiation (400 µmol photons m^{-2} s^{-1}).

Electron transport rate of photosystem II (ETR II): ETR(II) was monitored by using a Mini PAM photosynthesis yield analyzer (WALZ, Effeltrich, Germany). The measurements were performed on the last fully developed leaf, denoted as 'flag leaf' (Paul et al., 2016) in the 6th and 7th week after the start of the stress treatments. The apparent rate of electron transport was calculated as ETR(II) = Y(II) * PPFD * 0.5 * 0.84 (Genty et al., 1989), where Y(II) is the effective quantum yield of PSII, PPFD is the photon flux density of incident photosynthetically active radiation. The two coefficients (0.5 and 0.84) represent the fraction of absorbed light partitioned to PSII, and the probability that the

incident irradiance will be absorbed by PSII in higher plants, respectively (Björkman and Demmig, 1987; Schreiber, 2004).

Proline content determination: Fresh leaf samples (0.1g from the fully developed leaf below the flag leaf) were collected from all studied wheat cultivars and stored in liquid nitrogen. The content of free proline was determined as described earlier (Bates et al., 1973) at the 10th week after the start of the stress treatments. Samples were homogenized in 3% (w/v) sulfosalicylic acid to precipitate protein, and centrifuged at 14,000xg for 10 min. The reaction mixture contained 2 mL glacial acetic acid, 2 mL ninhydrin reagent (2.50 % w/v ninhydrin in 60 % v/v 6 M phosphoric acid) and 2 mL of the supernatant. The incubation lasted for 1 h at 90 °C then, after stopping the reaction with ice, 4 cm^3 of toluene was added and vortexed. The upper toluene phase was decanted into a glass cuvette and absorbance was measured at k = 520 nm. Each assay was performed in five replicates representing five leaves from different plants for each treatment. The content of proline was expressed as mg g^{-1} fresh weight according to a calibration curve with proline.

Soil properties: A mixture of Terra peat soil and sandy soil (3:1, v/v) was used for the experiments, with 0.9 kg/dm^3 specific gravity. Water retention curve was determined according to MSZ-08-0205:1978 by using pF box between at pF 0-2.5 (0.1 -31.6 kPa), pressure membrane extractor at pF 4.2 (1584.9 kPa) and vapor equilibrium technique above that. The measurements were performed by the accredited Soil Safety Laboratory of the National Food Chain Safety Office, Velence, Hungary.

Statistical analysis:
Calculation of mean and SD, tests for normal distribution of data, one-way ANOVA analysis of the significance level between mean differences, as well as heteroscedasticity tests for the distribution of residuals for the linear regression of data were performed by the XLSTAT-Premium software package (Addinsoft (2019), Boston, USA. https://www.xlstat.com).

References:

Bates, L.S., Waldren, R.P., Teare, I.D., 1973. Rapid determination of free proline for water-stress studies. Plant and Soil 39, 205-207.

Björkman, O., Demmig, B., 1987. Photon yield of O_2 evolution and chlorophyll fluorescence characteristics at 77 K among vascular plants of diverse origins. Planta 170, 489-504.

Genty, B., Briantais, J.-M., Baker, N.R., 1989. The relationship between the quantum yield of photosynthetic electron transport and quenching of chlorophyll fluorescence. Biochim. Biophys. Acta 990, 87-92.

Paul, K., Pauk, J., Deák, Z., Sass, L., Vass, I., 2016. Contrasting response of biomass and grain yield to severe drought in Cappelle Desprez and Plainsman V wheat cultivars. PeerJ 4, e1708; DOI 1710.7717/peerj.1708.

Schreiber, U., 2004. Pulse-Amplitude-Modulation (PAM) fluorometry and saturation pulse method: an overview, Chlorophyll a Fluorescence, Springer, Netherlands, pp. 279-319.

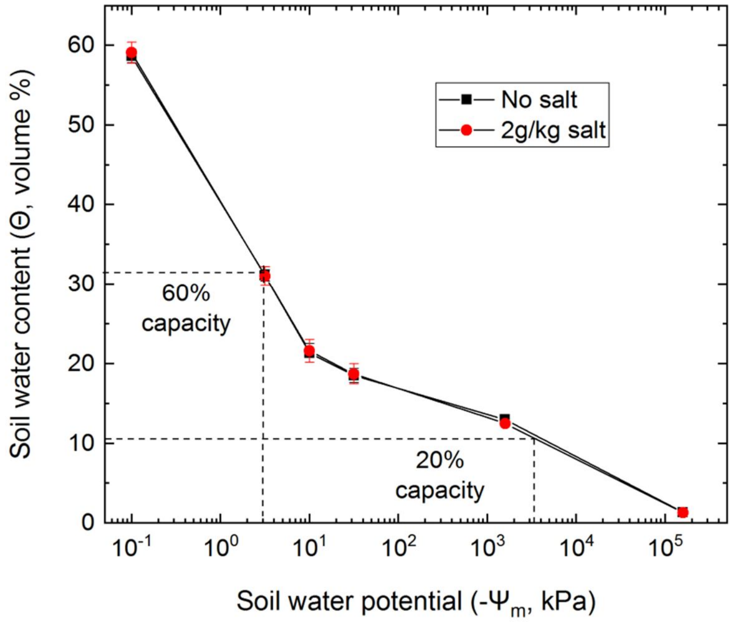

Supplementary Figure 1. Water retention curve of the soil used for the experiments. The measurements were performed in the 3:1 (V/V) mixture of peat and sand without addition, and in the presence of 2 g/kg NaCl. The data represent the means and SD of 5 different soil samples at pF 0, 1.5, 2.0 and 2.5. In case of the pF 4.2 and 6.2 points only one measurement of the no salt and of the 2g/kg samples was successful. The pF values are expressed in cm, that is why the values in kPa are 10-fold smaller. The dashed lines indicate 60% and 20% soil water capacity (considering the 0.9 kgL^{-1} specific gravity of the soil), which correspond to -3 (well watered) and -3500 kPa (water limited) soil water potential, respectively. Statistical analysis of the data is shown in Supplementary Table 1.

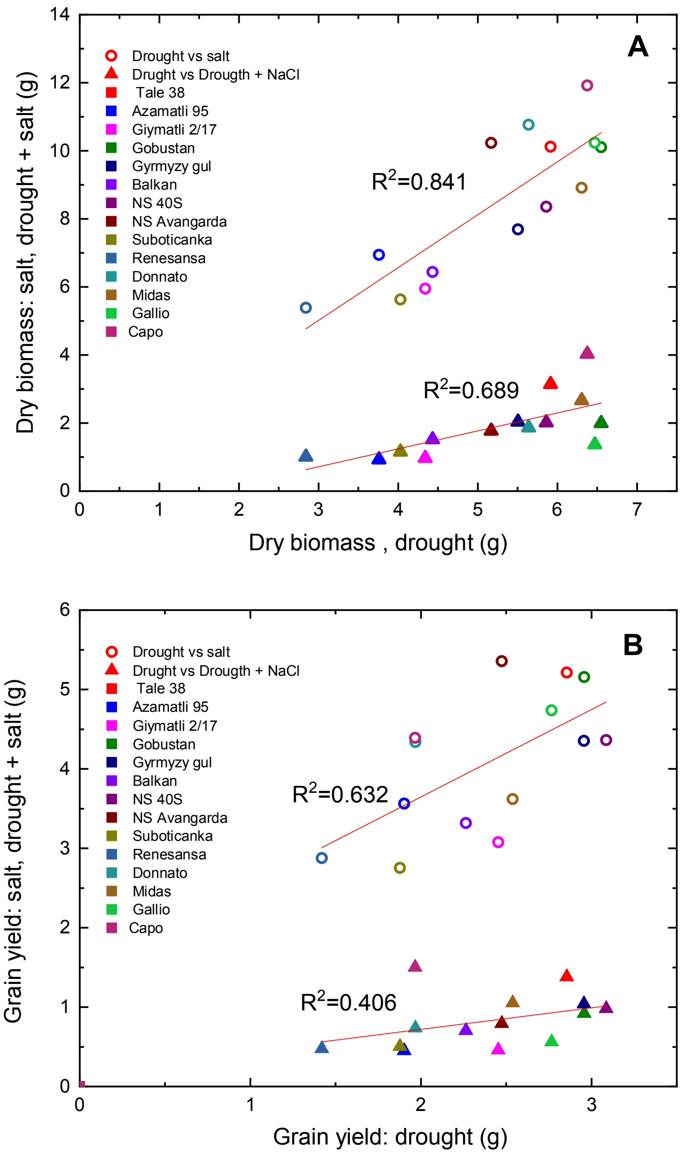

Supplementary Figure 2. Correlation of dry biomass and grain yield obtained under water limited versus saline and saline plus water limited conditions. A, Total dry biomass values obtained under well watered plus salt (T3), and water limited plus salt (T4) conditions are plotted as a function of total dry biomass obtained under water limited (T2) conditions. B, The same as A, but total grain yield is plotted. The shape of the symbols corresponds to the treatments, while the color code represents the different cultivars. Data shown are mean of n=5 plants/treatment. The red solid lines represent the best fitting linear correlation curves for each of the four treatments with the indicated Pearson's R^2 values.

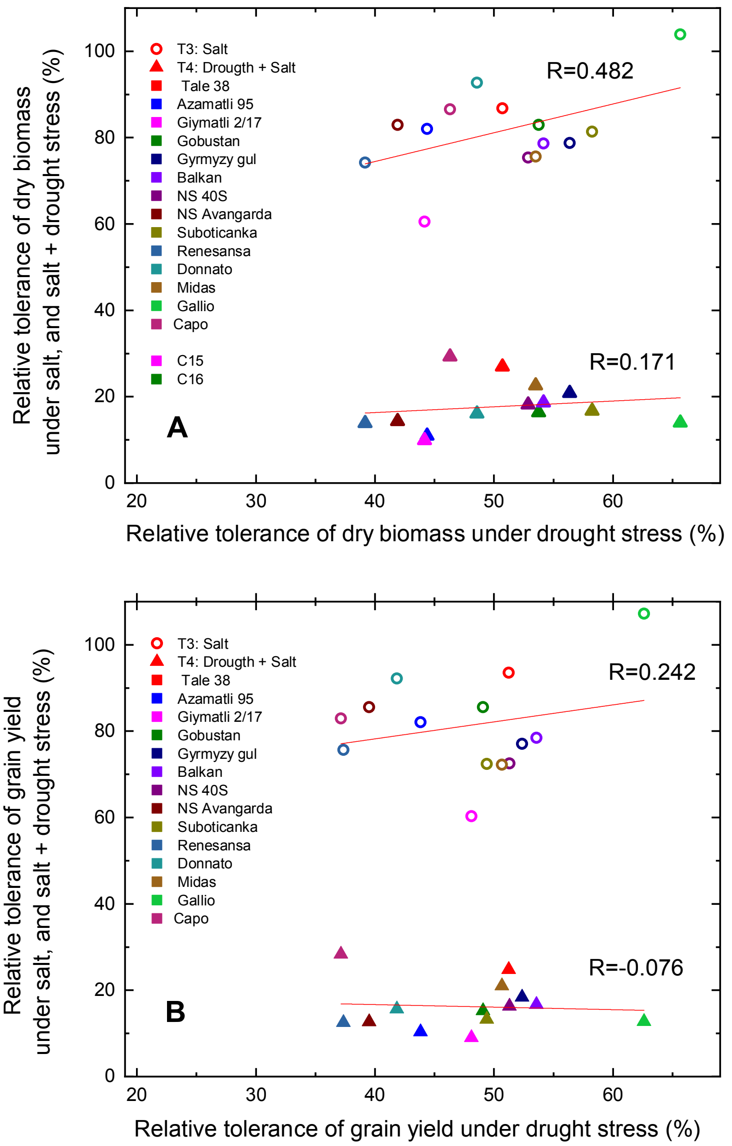

Supplementary Figure 3. Correlation of relative tolerance of dry biomass and grain yield obtained under water limited versus saline, and saline plus water limited conditions. Relative stress tolerance, or yield stability, was calculated as the ratio of total above ground biomass (or grain yield) obtained under stress and the well watered control conditions. A**,** Salinity tolerance of dry biomass is plotted as function of drought tolerance of dry biomass under well watered plus salt (T3), and water limited plus salt (T4) conditions. B, The same as A, but total grain yield tolerance values are plotted. The shape of the symbols corresponds to the treatments, while the color code represents the different cultivars. Data shown are mean of n=5 plants/treatment. The red solid lines represent the best fitting linear correlation curves for each of the four treatments with the indicated Pearson's R^2 values.

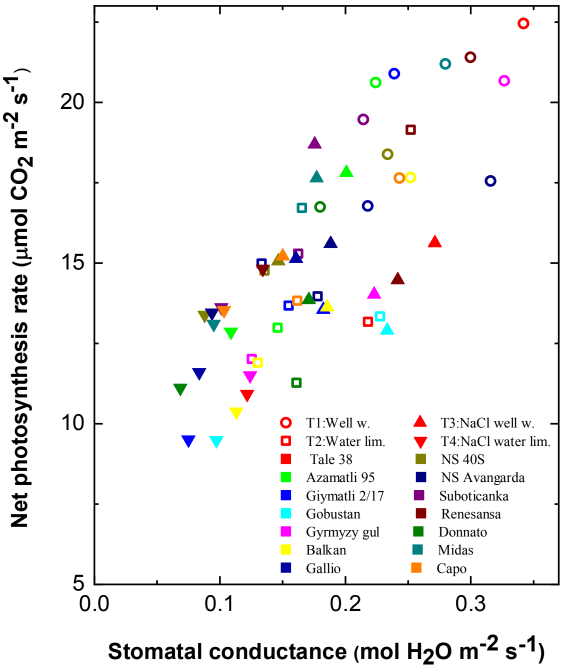

Supplementary Figure 4. Correlation of the net rate of photosynthesis with stomatal conductance. Net rate of photosynthesis (CO_2 uptake) is plotted as a function of stomatal conductance, both obtained from gas exchange measurements. The data obtained for the 14 selected wheat cultivars under well watered (T1), water limited (T2), salt plus well watered (T3), and salt plus water limited (T4) conditions. Data shown are mean± SE (n=5) plants/treatment.

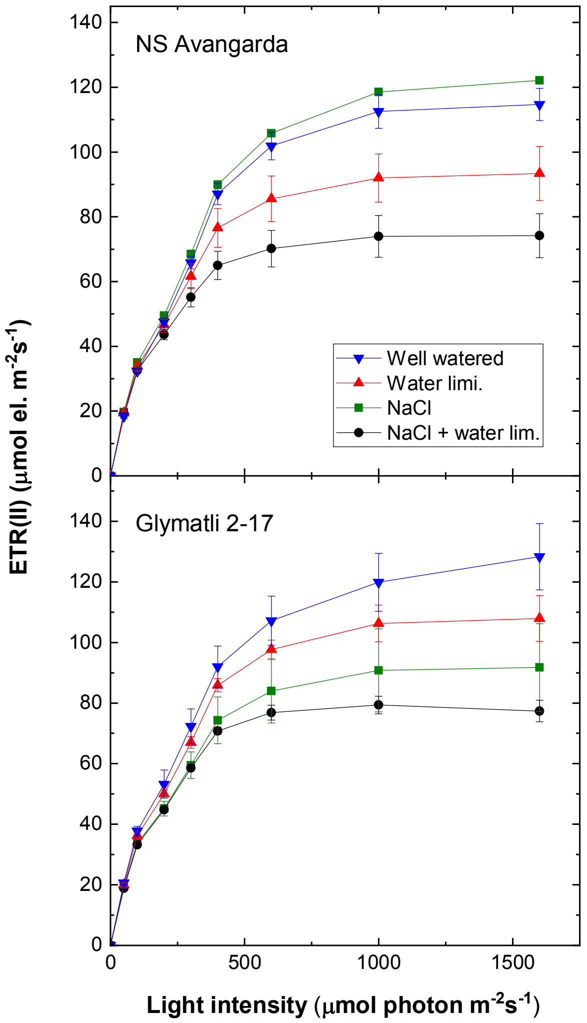

Supplementary Figure 5. Effect of salt and drought stress on the electron transport rate through Photosystem II. ETR(II) was determined as described in the Materials and Methods as a function of light intensity and shown for two selected cultivars. The measurements were performed under well watered (T1), water limited (T2), salt plus well watered (T3), and salt plus water limited (T4) conditions. Data shown are mean± SE (n=5) plants/treatment.

Supplementary Table 1. Data (% V/V) shown are the mean and SD of soil water content (n=5 samples/treatment). Calculation of mean and SD, tests for normal distribution of data, and one-way ANOVA analysis of the significance level between mean differences was performed by the XLSTAT software package. In the normality tests the p>0.05 values show normal distribution of data, while in the ANOVA table the p<0.05 show that the corresponding means are different at 5% significance level.

Treatmen	pF value	pF 0.0	pF 1.5	pF 2.0	pF 2.5	pF 4.2	pF 6.2
No add.	Mean	58.620	31.100	21.320	18.520	13.000	1.360
	Stand. dev.	0.817	0.711	1.182	0.876		
Normality tests	Shapiro-Wilk	0.106	0.117	0.585	0.805		
	Anderson-Darl	0.107	0.067	0.453	0.584		
	Lilliefors	0.317	**0.037**	0.326	0.412		
	Jarque-Bera	0.578	0.621	0.817	0.892		
NaCl	Mean	59.080	31.040	21.600	18.720	12.500	1.330
	Stand. dev.	1.308	1.155	1.437	1.274		
Normality tests	Shapiro-Wilk	0.697	0.421	0.240	0.102		
	Anderson-Darl	0.685	0.454	0.223	0.092		
	Lilliefors	0.846	0.581	0.192	0.128		
	Jarque-Bera	0.775	0.778	0.711	0.549		
Anova	Contrast	Pr > Diff	Pr > Diff	Pr > Diff	Pr > Diff		
	Noadd vs NaCl	0.524	0.924	0.745	0.780		

Supplementary Table 2. Heteroscedasticity tests for the distribution of residuals for the linear regression of data shown in Figures 4A, 4B, 5, and Supplementary Figures 2. and 3. The test were performed by the XLSTAT program package. p values larger than 0.05 show the cases where the hypothesis that residuals are homoscedastic cannot be rejected.

Heteroscedasticity tests: α=0.05, p-value (Two-tailed)				
Figure	Plot	Treatment	Breusch-Pagan	White
Fig. 4A	Dry biomass vs. Leaf area	T1	0.869	0.985
		T2	0.578	0.167
		T3	0.312	0.451
		T4	0.271	0.388
Fig. 4B	Grain yield vs. Leaf area	T1	0.779	0.843
		T2	0.644	0.371
		T3	0.153	0.232
		T4	0.055	0.077
Fig. 5.	Grain yield vs. Dry mass	T1	0.03	0.095
		T2	0.209	0.454
		T3	0.007	0.016
		T4	0.063	0.132
Suppl. Fig. 1.	Dry biomass	T3 vs. T2	0.505	0.232
		T4 vs T2	0.076	0.083
	Grain yield	T3 vs. T2	0.939	0.091
		T4 vs T2	0.571	0.602
Suppl. Fig. 2	Dry biomass tolerance	T3 vs. T2	0.954	0.807
		T4 vs T2	0.521	0.797
	Grain yield tolerance	T3 vs. T2	0.09	0.153
		T4 vs T2	0.143	0.285
Suppl. Fig. 3	Net photosynth rate vs. Stomatal conduct.	T1	0.937	0.415
		T2	0.976	0.327
		T3	0.830	0.290
		T4	0.361	0.558
		T1-T4	0.055	0.018

Supplementary Table 3. Effect of drought and salt stress on projected leaf area of wheat plants. Measurements were performed under well watered (T1), water limited (T2), salt plus well watered (T3), and salt plus water limited (T4) conditions. Data (cm^2) shown are the mean and SD (n=5 plants/treatment) of leaf area values obtained for the last three measurement days (in the 55-71 days range). Calculation of mean and SD, tests for normal distribution of data, and one-way ANOVA analysis of the significance level between mean differences was performed by the XLSTAT software package. In the normality tests the p>0.05 values show normal distribution of data, while in the ANOVA table the p<0.05 show that the corresponding means are different at 5% significance level.

Treatment	Cultivar	NS40S	NS Avang	Donnato	Midas	Gallio	Capo	Subotican	Tale 38	Renesans	Azamatli 9	Gymatli 2/	Gobustan	Gyrmyzy g	Balkan
T1	Mean	110.8	134.7	156.6	147.6	116.7	175.1	81.6	133.9	78.8	83.7	110.2	131.4	90.5	89.3
	Standard deviation	16.1	19.5	15.6	12.3	8.4	9.9	12.4	15.7	11.1	21.0	12.4	17.4	13.2	9.6
Normality tests	Shapiro-Wilk	0.786	0.637	0.362	0.990	0.993	0.733	0.532	0.051	0.934	0.814	0.457	0.549	0.306	0.798
	Anderson-Darling	0.675	0.370	0.334	0.954	0.977	0.790	0.449	0.068	0.894	0.901	0.278	0.498	0.406	0.796
	Lilliefors	0.352	0.180	0.219	0.859	0.997	0.866	0.584	0.104	0.833	0.977	0.513	0.266	0.434	0.853
	Jarque-Bera	0.769	0.749	0.580	0.894	0.920	0.854	0.722	0.143	0.755	0.707	0.800	0.647	0.580	0.758
T2	Mean	62.6	64.7	86.2	86.5	76.1	102.5	49.5	65.4	35.0	35.2	47.7	74.2	64.3	48.0
	Standard deviation	12.7	12.8	27.4	8.9	19.3	21.5	9.8	13.2	9.3	4.4	10.6	13.5	8.1	9.4
Normality tests	Shapiro-Wilk	0.854	0.946	0.291	0.398	0.226	0.353	0.354	0.971	0.164	0.701	0.404	0.893	0.896	**0.023**
	Anderson-Darling	0.718	0.953	0.405	0.508	0.192	0.269	0.591	0.906	0.242	0.704	0.514	0.846	0.907	**0.031**
	Lilliefors	0.530	0.982	0.803	0.388	0.050	0.377	0.879	0.643	0.388	0.662	0.888	0.877	0.853	0.193
	Jarque-Bera	0.837	0.730	0.549	0.616	0.640	0.616	0.689	0.800	0.528	0.784	0.576	0.729	0.749	0.215
T3	Mean	95.3	133.4	171.5	130.5	128.0	168.6	83.2	121.5	65.5	80.0	72.5	112.5	88.4	71.6
	Standard deviation	14.1	8.5	11.5	13.5	6.3	6.5	8.9	10.3	9.0	18.5	10.1	9.3	10.9	7.5
Normality tests	Shapiro-	0.073	0.167	0.586	0.977	0.175	0.159	0.471	0.863	0.298	0.445	0.395	0.251	0.948	0.323
	Anderson-Darling	0.096	0.257	0.583	0.966	0.276	0.117	0.534	0.824	0.229	0.322	0.377	0.403	0.948	0.388
	Lilliefors	0.152	0.570	0.427	0.959	0.240	0.105	0.765	0.802	0.180	0.148	0.308	0.766	0.945	0.241
	Jarque-Bera	0.468	0.550	0.639	0.739	0.519	0.859	0.675	0.724	0.560	0.598	0.627	0.583	0.733	0.571
T4	Mean	26.7	25.6	30.5	42.7	21.6	66.5	17.7	44.9	13.1	12.5	13.1	23.2	27.9	20.4
	Standard deviation	2.5	6.7	15.9	16.8	10.8	6.0	8.2	12.9	4.3	6.5	2.4	6.2	6.3	2.9
Normality tests	Shapiro-Wilk	0.801	0.237	0.487	0.265	0.513	0.778	0.060	0.297	0.091	0.090	0.760	0.541	0.498	0.451
	Anderson-Darling	0.834	0.254	0.647	0.296	0.539	0.592	0.087	0.417	0.138	0.112	0.642	0.270	0.442	0.378
	Lilliefors	0.805	0.329	0.755	0.555	0.777	0.536	0.227	0.526	0.199	0.204	0.485	0.110	0.546	0.601
	Jarque-Bera	0.711	0.600	0.658	0.645	0.809	0.731	0.500	0.593	0.526	0.526	0.719	0.820	0.712	0.589
ANOVA	Contrast	Pr > Diff	Pr > Diff	Pr > Diff	Pr > Diff	Pr > Diff	Pr > Diff	Pr > Diff	Pr > Diff	Pr > Diff	Pr > Diff	Pr > Diff	Pr > Diff	Pr > Diff	Pr > Diff
	T1 vs T4	< 0.0001	< 0.0001	< 0.0001	< 0.0001	< 0.0001	< 0.0001	< 0.0001	< 0.0001	< 0.0001	< 0.0001	< 0.0001	< 0.0001	< 0.0001	< 0.0001
	T1 vs T2	< 0.0001	< 0.0001	< 0.0001	< 0.0001	< 0.0001	< 0.0001	< 0.0001	< 0.0001	< 0.0001	< 0.0001	< 0.0001	< 0.0001	< 0.0001	< 0.0001
	T1 vs T3	0.006	**0.981**	**0.138**	0.007	0.025	**0.505**	**0.973**	**0.057**	0.001	**0.991**	< 0.0001	0.000	**0.937**	< 0.0001
	T3 vs T4	< 0.0001	< 0.0001	< 0.0001	< 0.0001	< 0.0001	< 0.0001	< 0.0001	< 0.0001	< 0.0001	< 0.0001	< 0.0001	< 0.0001	< 0.0001	< 0.0001
	T3 vs T2	< 0.0001	< 0.0001	< 0.0001	< 0.0001	< 0.0001	< 0.0001	< 0.0001	< 0.0001	< 0.0001	< 0.0001	< 0.0001	< 0.0001	< 0.0001	< 0.0001
	T2 vs T4	< 0.0001	< 0.0001	< 0.0001	< 0.0001	< 0.0001	< 0.0001	< 0.0001	0.000	< 0.0001	0.001	< 0.0001	< 0.0001	< 0.0001	< 0.0001

Supplementary Table 4. Effect of drought and salt stress on dry biomass of wheat plants. Measurements were performed under well watered (T1), water limited (T2), salt plus well watered (T3), and salt plus water limited (T4) conditions. Data (g) shown are the mean and SD (n=5 plants/treatment). Calculation of mean and SD, tests for normal distribution of data, and one-way ANOVA analysis of the significance level between mean differences was performed by the XLSTAT software package. In the normality tests the $p>0.05$ values show normal distribution of data, while in the ANOVA table the $p<0.05$ show that the corresponding means are different at 5% significance level.

Treatment	Cultivar	Tale 38	Azamatli	Giymatli 2	Gobustan	Gyrmyzy	Balkan	NS 40S	NS Avang	Suboticar	Renesans	Donnato	Midas	Gallio	Capo
T1	Mean	11.662	8.466	9.826	12.184	9.768	8.182	11.086	12.338	6.916	7.256	11.612	11.788	9.856	13.770
	Standard deviation	1.257	1.550	0.365	1.383	0.555	0.838	1.703	0.977	0.308	0.883	0.778	1.059	1.143	1.358
Normality tests	Shapiro-Wilk	0.641	0.096	**0.027**	0.556	**0.022**	0.340	0.212	**0.027**	0.311	0.202	0.490	0.532	0.340	**0.045**
	Anderson-Darling	0.430	0.106	**0.037**	0.286	**0.024**	0.296	0.208	**0.020**	0.262	0.190	0.342	0.323	0.285	**0.045**
	Lilliefors	0.281	0.222	0.075	0.272	**0.012**	0.343	0.212	**0.006**	0.224	0.199	0.242	0.148	0.265	**0.034**
	Jarque-Bera	0.847	0.690	0.664	0.970	0.468	0.743	0.711	0.506	0.679	0.702	0.750	0.841	0.752	0.500
T2	Mean	5.914	3.758	4.340	6.550	5.506	4.432	5.862	5.170	4.028	2.842	5.640	6.306	6.472	6.376
	Standard deviation	0.847	0.416	0.801	0.371	0.435	1.057	0.658	0.607	0.289	0.613	1.906	0.541	1.174	0.712
Normality tests	Shapiro-Wilk	0.633	0.135	0.978	0.445	0.542	0.580	0.147	0.186	0.737	**0.037**	0.252	0.097	0.465	0.597
	Anderson-Darling	0.423	0.132	0.883	0.403	0.535	0.548	0.141	0.166	0.622	**0.046**	0.167	0.092	0.384	0.575
	Lilliefors	0.269	0.122	0.928	0.403	0.655	0.698	0.152	0.232	0.689	0.072	0.091	0.137	0.618	0.776
	Jarque-Bera	0.844	0.709	0.898	0.738	0.757	0.769	0.692	0.727	0.831	0.668	0.655	0.550	0.830	0.760
T3	Mean	10.118	6.942	5.950	10.104	7.692	6.434	8.358	10.232	5.628	5.386	10.766	8.914	10.240	11.918
	Standard deviation	0.808	1.311	0.665	0.616	1.191	0.871	1.100	0.581	0.472	0.575	1.648	0.783	0.876	1.125
Normality tests	Shapiro-Wilk	0.736	0.237	0.287	0.987	0.120	0.747	0.167	0.803	0.874	0.387	0.994	0.354	0.506	0.835
	Anderson-Darling	0.737	0.201	0.233	0.877	0.123	0.605	0.149	0.797	0.775	0.268	0.932	0.230	0.487	0.683
	Lilliefors	0.829	0.305	0.129	0.933	0.304	0.474	0.272	0.801	0.826	0.179	0.994	0.125	0.615	0.578
	Jarque-Bera	0.782	0.646	0.725	0.931	0.616	0.809	0.619	0.804	0.858	0.705	0.872	0.710	0.750	0.855
T4	Mean	3.142	0.926	0.970	1.994	2.038	1.522	2.014	1.770	1.156	1.004	1.866	2.664	1.374	4.028
	Standard deviation	0.827	0.614	0.187	0.516	0.429	0.354	0.159	0.737	0.581	0.307	1.169	1.124	0.933	0.620
Normality tests	Shapiro-Wilk	0.400	0.654	0.326	0.903	0.708	0.358	0.631	0.295	0.224	0.241	0.405	0.323	0.814	0.785
	Anderson-Darling	0.336	0.518	0.268	0.708	0.538	0.325	0.610	0.290	0.219	0.203	0.325	0.300	0.570	0.649
	Lilliefors	0.385	0.464	0.290	0.799	0.713	0.451	0.824	0.365	0.346	0.172	0.320	0.508	0.402	0.713
	Jarque-Bera	0.773	0.835	0.760	0.922	0.808	0.769	0.788	0.725	0.720	0.712	0.713	0.787	0.921	0.814
ANOVA	Contrast	Pr > Diff	Pr > Diff	Pr > Diff	Pr > Diff	Pr > Diff	Pr > Diff	Pr > Diff	Pr > Diff	Pr > Diff	Pr > Diff	Pr > Diff	Pr > Diff	Pr > Diff	Pr > Diff
	T1 vs T4	< 0.0001	< 0.0001	< 0.0001	< 0.0001	< 0.0001	< 0.0001	< 0.0001	< 0.0001	< 0.0001	< 0.0001	< 0.0001	< 0.0001	< 0.0001	< 0.0001
	T1 vs T2	< 0.0001	< 0.0001	< 0.0001	< 0.0001	< 0.0001	< 0.0001	< 0.0001	< 0.0001	< 0.0001	< 0.0001	< 0.0001	< 0.0001	0.000	< 0.0001
	T1 vs T3	**0.088**	**0.157**	< 0.0001	0.005	0.002	0.019	0.005	0.002	0.002	0.001	**0.791**	0.001	**0.935**	0.044
	T3 vs T4	< 0.0001	< 0.0001	< 0.0001	< 0.0001	< 0.0001	< 0.0001	< 0.0001	< 0.0001	< 0.0001	< 0.0001	< 0.0001	< 0.0001	< 0.0001	< 0.0001
	T3 vs T2	< 0.0001	0.001	0.002	< 0.0001	0.001	0.007	0.010	< 0.0001	< 0.0001	< 0.0001	0.000	0.002	0.001	< 0.0001
	T2 vs T4	0.002	0.004	< 0.0001	< 0.0001	< 0.0001	0.000	0.000	< 0.0001	< 0.0001	0.001	0.004	< 0.0001	< 0.0001	0.009

Supplementary Table 5. Effect of drought and salt stress on grain yield of wheat plants. Measurements were performed under well watered (T1), water limited (T2), salt plus well watered (T3), and salt plus water limited (T4) conditions. Data (g) shown are the mean and SD (n=5 plants/treatment). Calculation of mean and SD, tests for normal distribution of data, and one-way ANOVA analysis of the significance level between mean differences was performed by the XLSTAT software package. In the normality tests the p>0.05 values show normal distribution of data, while in the ANOVA table the p<0.05 show that the corresponding means are different at 5% significance level.

Treatment	Cultivar	Tale 38	Azamatli	Giymatli 2	Gobustan	Gyrmyzy	Balkan	NS 40S	NS Avang	Suboticar	Renesans	Donnato	Midas	Gallio	Capo
T1	Mean	5.574	4.340	5.102	6.026	5.646	4.226	6.016	6.260	3.802	3.802	4.704	5.010	4.418	5.294
	Standard deviation	0.453	0.728	0.223	0.720	0.384	0.464	0.990	0.601	0.628	0.488	0.347	0.635	0.640	0.888
Normality tests	Shapiro-Wilk	0.912	0.161	0.510	0.512	0.050	0.445	0.327	0.108	0.355	**0.028**	0.368	0.728	0.316	0.135
	Anderson-Darling	0.808	0.157	0.477	0.504	0.060	0.362	0.315	0.108	0.161	**0.035**	0.301	0.453	0.260	0.092
	Lilliefors	0.786	0.149	0.544	0.606	0.161	0.232	0.350	0.121	0.108	0.051	0.268	0.510	0.195	**0.033**
	Jarque-Bera	0.867	0.709	0.748	0.761	0.584	0.748	0.726	0.683	0.896	0.664	0.766	0.962	0.701	0.597
T2	Mean	2.856	1.902	2.454	2.958	2.956	2.264	3.086	2.474	1.878	1.420	2.460	2.538	2.766	1.966
	Standard deviation	0.473	0.321	0.490	0.259	0.158	0.575	0.328	0.328	0.161	0.341	0.397	0.215	0.490	0.517
Normality tests	Shapiro-Wilk	0.681	0.712	0.926	0.773	0.994	0.738	0.276	0.840	0.526	0.390	0.112	**0.011**	0.391	0.642
	Anderson-Darling	0.644	0.574	0.825	0.724	0.939	0.692	0.244	0.748	0.349	0.332	0.084	**0.016**	0.339	0.600
	Lilliefors	0.667	0.396	0.724	0.808	0.999	0.827	0.209	0.900	0.220	0.269	**0.037**	**0.034**	0.356	0.874
	Jarque-Bera	0.796	0.801	0.858	0.795	0.865	0.791	0.713	0.856	0.787	0.735	0.569	0.450	0.755	0.790
T3	Mean	5.212	3.562	3.076	5.156	4.352	3.316	4.362	5.354	2.752	2.876	4.336	3.618	4.736	4.390
	Standard deviation	0.759	0.528	0.408	0.567	0.748	0.535	0.548	0.404	0.256	0.228	1.119	0.388	0.503	0.693
Normality tests	Shapiro-Wilk	0.404	0.286	0.583	**0.018**	0.249	0.395	0.375	0.667	0.881	0.461	0.897	0.818	0.059	0.950
	Anderson-Darling	0.410	0.288	0.522	**0.022**	0.213	0.381	0.354	0.664	0.849	0.490	0.801	0.729	0.065	0.861
	Lilliefors	0.590	0.692	0.464	**0.013**	0.273	0.526	0.538	0.799	0.925	0.631	0.861	0.666	0.099	0.905
	Jarque-	0.754	0.697	0.761	0.468	0.631	0.726	0.708	0.773	0.814	0.762	0.843	0.811	0.631	0.872
T4	Mean	1.382	0.450	0.456	0.920	1.040	0.706	0.982	0.794	0.504	0.476	0.736	1.052	0.562	1.502
	Standard deviation	0.462	0.316	0.074	0.272	0.270	0.218	0.074	0.426	0.297	0.143	0.450	0.448	0.367	0.316
Normality tests	Shapiro-Wilk	0.595	0.843	0.310	0.133	0.417	0.539	0.228	0.168	0.074	0.424	0.417	0.915	0.631	0.877
	Anderson-Darling	0.522	0.802	0.282	0.065	0.346	0.546	0.220	0.137	0.081	0.391	0.329	0.763	0.390	0.700
	Lilliefors	0.549	0.877	0.405	**0.022**	0.543	0.625	0.342	0.092	0.173	0.654	0.380	0.664	0.239	0.827
	Jarque-Bera	0.773	0.837	0.790	0.697	0.693	0.764	0.727	0.701	0.557	0.812	0.693	0.900	0.893	0.885
ANOVA	Contrast	Pr > Diff	Pr > Diff	Pr > Diff	Pr > Diff	Pr > Diff	Pr > Diff	Pr > Diff	Pr > Diff	Pr > Diff	Pr > Diff	Pr > Diff	Pr > Diff	Pr > Diff	Pr > Diff
	T1 vs T4	< 0.0001	< 0.0001	< 0.0001	< 0.0001	< 0.0001	< 0.0001	< 0.0001	< 0.0001	< 0.0001	< 0.0001	< 0.0001	< 0.0001	< 0.0001	< 0.0001
	T1 vs T2	< 0.0001	< 0.0001	< 0.0001	< 0.0001	< 0.0001	< 0.0001	< 0.0001	< 0.0001	< 0.0001	< 0.0001	0.001	< 0.0001	< 0.0001	< 0.0001
	T1 vs T3	**0.731**	**0.108**	< 0.0001	**0.059**	0.002	0.033	0.002	0.027	0.002	0.002	0.822	0.001	**0.758**	**0.156**
	T3 vs T4	< 0.0001	< 0.0001	< 0.0001	< 0.0001	< 0.0001	< 0.0001	< 0.0001	< 0.0001	< 0.0001	< 0.0001	< 0.0001	< 0.0001	< 0.0001	< 0.0001
	T3 vs T2	< 0.0001	0.000	0.047	< 0.0001	0.001	0.013	0.017	< 0.0001	0.010	< 0.0001	0.004	0.007	0.001	0.000
	T2 vs T4	0.003	0.002	< 0.0001	< 0.0001	< 0.0001	0.000	0.000	0.000	0.000	0.002	0.008	0.000	< 0.0001	**0.667**

Supplementary Table 6. Effect of drought and salt stress on harvest index of wheat plants. Measurements were performed under well watered (T1), water limited (T2), salt plus well watered (T3), and salt plus water limited (T4) conditions. Data shown are the mean and SD (n=5 plants/treatment). Calculation of mean and SD, tests for normal distribution of data, and one-way ANOVA analysis of the significance level between mean differences was performed by the XLSTAT software package. In the normality tests the $p>0.05$ values show normal distribution of data, while in the ANOVA table the $p<0.05$ show that the corresponding means are different at 5% significance level.

Treatment	Cultivar	Tale 38	Azamatli	Giymatli 2	Gobustan	Gyrmyzy	Balkan	NS 40S	NS Avang	Subotican	Renesans	Donnato	Midas	Gallio	Capo
T1	Mean	0.480	0.514	0.519	0.496	0.578	0.516	0.542	0.508	0.549	0.524	0.405	0.424	0.447	0.383
	Standard deviation	0.037	0.027	0.007	0.037	0.011	0.017	0.007	0.040	0.084	0.013	0.007	0.020	0.016	0.034
Normality tests	Shapiro-Wilk	0.706	0.174	0.562	0.855	0.583	0.748	0.840	**0.020**	0.115	0.850	0.253	0.167	0.986	0.516
	Anderson-Darling	0.615	0.151	0.510	0.836	0.561	0.636	0.791	**0.024**	0.072	0.727	0.156	0.142	0.877	0.334
	Lilliefors	0.692	0.119	0.658	0.922	0.789	0.636	0.810	**0.020**	**0.028**	0.757	0.062	0.096	0.944	0.161
	Jarque-Bera	0.805	0.633	0.759	0.810	0.763	0.814	0.810	0.464	0.594	0.870	0.680	0.691	0.927	0.799
T2	Mean	0.483	0.504	0.564	0.451	0.538	0.509	0.527	0.478	0.466	0.499	0.381	0.403	0.428	0.308
	Standard deviation	0.034	0.039	0.015	0.024	0.023	0.012	0.016	0.017	0.016	0.040	0.018	0.008	0.017	0.069
Normality tests	Shapiro-Wilk	0.957	0.346	0.682	0.430	0.920	0.929	0.847	0.539	0.742	0.094	0.390	0.773	0.123	0.083
	Anderson-Darling	0.788	0.219	0.455	0.334	0.765	0.727	0.637	0.473	0.637	0.091	0.348	0.520	0.092	0.086
	Lilliefors	0.869	0.133	0.349	0.411	0.868	0.778	0.634	0.524	0.692	0.137	0.407	0.470	0.068	0.163
	Jarque-Bera	0.887	0.760	0.863	0.756	0.864	0.896	0.867	0.780	0.809	0.676	0.790	0.893	0.682	0.681
T3	Mean	0.514	0.516	0.517	0.510	0.565	0.514	0.522	0.523	0.492	0.536	0.397	0.406	0.462	0.367
	Standard deviation	0.043	0.024	0.047	0.033	0.015	0.018	0.009	0.013	0.063	0.033	0.053	0.022	0.013	0.023
Normality tests	Shapiro-Wilk	0.890	0.852	0.961	0.194	0.480	0.713	0.443	0.403	0.765	0.677	**0.031**	0.173	0.877	0.994
	Anderson-Darling	0.706	0.821	0.901	0.198	0.505	0.465	0.439	0.420	0.518	0.399	**0.038**	0.140	0.643	0.935
	Lilliefors	0.690	0.835	0.948	0.324	0.635	0.353	0.472	0.652	0.432	0.308	0.169	0.163	0.702	0.997
	Jarque-Bera	0.902	0.819	0.844	0.680	0.788	0.915	0.811	0.783	0.913	0.964	0.515	0.641	0.941	0.872
T4	Mean	0.424	0.479	0.476	0.478	0.505	0.448	0.504	0.417	0.428	0.471	0.400	0.402	0.414	0.370
	Standard deviation	0.040	0.062	0.018	0.043	0.048	0.045	0.016	0.066	0.051	0.023	0.043	0.040	0.030	0.027
Normality tests	Shapiro-Wilk	0.791	**0.027**	0.276	0.463	0.473	0.730	0.234	0.220	0.944	0.114	0.928	0.436	0.591	0.819
	Anderson-Darling	0.639	**0.035**	0.258	0.410	0.287	0.530	0.165	0.208	0.805	0.096	0.751	0.319	0.511	0.560
	Lilliefors	0.808	**0.039**	0.314	0.437	0.174	0.488	0.125	0.274	0.943	0.093	0.811	0.299	0.680	0.547
	Jarque-Bera	0.828	0.637	0.788	0.794	0.816	0.836	0.712	0.749	0.864	0.673	0.879	0.758	0.782	0.898
ANOVA	Contrast	Pr > Diff	Pr > Diff	Pr > Diff	Pr > Diff	Pr > Diff	Pr > Diff	Pr > Diff	Pr > Diff	Pr > Diff	Pr > Diff	Pr > Diff	Pr > Diff	Pr > Diff	Pr > Diff
	T1 vs T2	1.000	0.977	0.075	0.274	0.109	0.974	0.656	0.651	0.142	0.533	0.752	0.491	0.390	0.051
	T1 vs T3	0.529	1.000	1.000	0.931	0.855	0.999	0.451	0.939	0.418	0.912	0.987	0.617	0.622	0.923
	T1 vs T4	0.244	0.546	0.061	0.517	**0.002**	**0.013**	**0.004**	**0.041**	**0.021**	0.066	0.996	0.344	0.089	0.960
	T2vs T3	0.593	0.963	0.062	0.102	0.383	0.991	0.985	0.333	0.890	0.219	0.901	0.996	0.052	0.158
	T2 vs T4	0.206	0.767	**0.000**	0.962	0.219	**0.030**	**0.043**	0.316	0.739	0.550	0.880	0.992	0.737	0.126
	T3 vs T4	**0.020**	0.506	0.073	0.229	**0.010**	**0.017**	0.083	**0.013**	0.339	**0.018**	1.000	0.959	**0.010**	0.999

Supplementary Table 7. Effect of drought and salt stress on water use of wheat plants. Measurements were performed under well watered (T1), water limited (T2), salt plus well watered (T3), and salt plus water limited (T4) conditions. Data for the total amount (mL) of water used during the experiment shown are the mean and SD (n=5 plants/treatment). Calculation of mean and SD, tests for normal distribution of data, and one-way ANOVA analysis of the significance level between mean differences was performed by the XLSTAT software package. In the normality tests the p>0.05 values show normal distribution of data, while in the ANOVA table the p<0.05 show that the corresponding means are different at 5% significance level.

Treatment	Cultivar	Tale 38	Azamatli 9	Giymatli 2	Gobustan	Gyrmyzy	Balkan	NS 40S	NS Avanga	Subotican	Renesansa	Donnato	Midas	Gallio	Capo
T1	Mean	3152.8	2352.8	2369.8	2908.4	2802.0	2401.4	2610.6	3102.4	2009.2	2226.4	2966.2	2434.2	2313.0	3038.0
	Standard deviation	367.5	159.5	89.9	285.7	46.9	437.3	609.8	191.3	254.8	217.8	237.1	114.5	179.9	389.3
Normality tests	Shapiro-Wilk	0.860	0.878	**0.006**	0.110	0.208	0.263	0.991	0.166	0.202	0.839	0.933	0.485	0.155	0.136
	Anderson-Darling	0.606	0.706	**0.010**	0.104	0.195	0.220	0.930	0.119	0.095	0.821	0.777	0.425	0.131	0.100
	Lilliefors	0.560	0.714	**0.009**	0.089	0.389	0.290	0.990	0.083	**0.040**	0.824	0.836	0.342	0.148	0.120
	Jarque-Bera	0.954	0.900	0.426	0.679	0.628	0.764	0.866	0.596	0.764	0.820	0.909	0.746	0.641	0.580
T2	Mean	1329.0	887.4	975.4	1386.2	1199.4	1007.8	1200.4	1034.8	893.6	775.2	1144.4	1274.4	1376.0	1372.6
	Standard deviation	217.2	43.4	190.0	97.6	100.6	304.3	115.8	136.9	74.5	158.8	358.4	166.2	231.7	171.6
Normality tests	Shapiro-Wilk	0.904	0.678	0.887	0.223	0.896	0.526	0.178	0.330	0.995	0.102	0.181	0.289	0.472	0.844
	Anderson-Darling	0.769	0.477	0.843	0.175	0.674	0.532	0.170	0.361	0.912	0.101	0.150	0.162	0.478	0.741
	Lilliefors	0.770	0.336	0.875	0.104	0.655	0.689	0.149	0.437	0.985	0.091	0.108	0.093	0.481	0.822
	Jarque-Bera	0.874	0.845	0.836	0.650	0.945	0.754	0.713	0.749	0.905	0.689	0.619	0.726	0.795	0.855
T3	Mean	2928.6	2175.2	1710.2	2647.2	2191.2	1937.2	1891.4	2404.6	1708.6	1797.4	2287.8	2165.2	2719.2	2969.4
	Standard deviation	280.2	256.1	173.5	186.7	394.6	366.2	290.2	150.4	271.4	131.1	241.9	178.0	147.1	169.7
Normality tests	Shapiro-Wilk	0.599	0.184	0.393	0.905	0.866	0.327	**0.013**	0.657	0.815	0.393	0.198	0.608	0.117	0.502
	Anderson-Darling	0.523	0.162	0.405	0.741	0.661	0.292	**0.014**	0.470	0.766	0.375	0.174	0.493	0.118	0.442
	Lilliefors	0.432	0.123	0.652	0.698	0.838	0.239	**0.007**	0.619	0.843	0.382	0.125	0.368	0.102	0.409
	Jarque-	0.764	0.691	0.757	0.895	0.902	0.724	0.448	0.825	0.806	0.742	0.706	0.776	0.705	0.753
T4	Mean	785.4	402.8	197.6	597.8	556.2	437.4	512.8	471.2	395.8	391.4	490.6	609.0	423.4	818.4
	Standard deviation	162.4	105.4	66.2	81.5	77.6	58.0	25.5	106.9	93.1	46.9	183.1	202.7	149.2	137.4
Normality tests	Shapiro-Wilk	0.426	0.220	0.838	0.726	0.805	0.476	0.874	0.412	0.927	0.926	0.228	0.370	0.492	0.579
	Anderson-Darling	0.454	0.227	0.770	0.726	0.771	0.453	0.784	0.360	0.843	0.765	0.211	0.334	0.319	0.535
	Lilliefors	0.629	0.290	0.816	0.744	0.880	0.548	0.655	0.266	0.911	0.900	0.169	0.549	0.187	0.833
	Jarque-Bera	0.780	0.728	0.814	0.793	0.804	0.825	0.830	0.737	0.856	0.909	0.718	0.827	0.780	0.767
ANOVA	Contrast	Pr > Diff	Pr > Diff	Pr > Diff	Pr > Diff	Pr > Diff	Pr > Diff	Pr > Diff	Pr > Diff	Pr > Diff	Pr > Diff	Pr > Diff	Pr > Diff	Pr > Diff	Pr > Diff
	T1 vs T4	< 0.0001	< 0.0001	< 0.0001	< 0.0001	< 0.0001	< 0.0001	< 0.0001	< 0.0001	< 0.0001	< 0.0001	< 0.0001	< 0.0001	< 0.0001	< 0.0001
	T1 vs T2	< 0.0001	< 0.0001	< 0.0001	< 0.0001	< 0.0001	< 0.0001	< 0.0001	< 0.0001	< 0.0001	< 0.0001	0.001	< 0.0001	< 0.0001	< 0.0001
	T1 vs T3	**0.731**	**0.108**	< 0.0001	**0.059**	0.002	0.033	0.002	0.027	0.002	0.002	**0.822**	0.001	**0.758**	**0.156**
	T3 vs T4	< 0.0001	< 0.0001	< 0.0001	< 0.0001	< 0.0001	< 0.0001	< 0.0001	< 0.0001	< 0.0001	< 0.0001	< 0.0001	< 0.0001	< 0.0001	< 0.0001
	T3 vs T2	< 0.0001	0.000	0.047	< 0.0001	0.001	0.013	0.017	< 0.0001	0.010	< 0.0001	0.004	0.007	0.001	0.000
	T2 vs T4	0.003	0.002	< 0.0001	< 0.0001	< 0.0001	0.000	0.000	0.000	0.000	0.002	0.008	0.000	< 0.0001	**0.667**

Supplementary Table 8. Effect of drought and salt stress on net photosynthesis rate in wheat plants. Gas exchange measurements were performed by a Licor 6400 gas analyzer under well watered (T1), water limited (T2), salt plus well watered (T3), and salt plus water limited (T4) conditions. Data (μmol CO_2 m^{-2} s^{-1}) shown are the mean and SD (n=12-14 measurements). Calculation of mean and SD, tests for normal distribution of data, and one-way ANOVA analysis of the significance level between mean differences was performed by the XLSTAT software package. In the normality tests the $p>0.05$ values show normal distribution of data, while in the ANOVA table the $p<0.05$ show that the corresponding means are different at 5% significance level.

Treatment	Cultivar	Tale 38	Azamatli 9	Giymatli 2	Gobustan	Gyrmyzy g	Balkan	NS 40S	NS Avanga	Subotican	Renesans	Donnato	Midas	Gallio	Capo
T1	Mean	22.45	20.62	20.89	21.65	20.67	17.66	18.38	17.55	19.46	21.40	16.75	21.19	16.78	17.65
	Standard deviation	0.98	1.10	0.26	1.51	2.29	0.68	0.33	0.46	0.47	1.10	0.35	0.43	0.24	1.07
Normality tests	Shapiro-Wilk	0.197	0.022	0.926	0.003	0.003	0.103	0.517	0.004	0.088	0.009	0.518	0.705	0.188	0.003
	Anderson-Darling	0.340	0.011	0.671	0.001	0.001	0.076	0.272	0.003	0.105	0.006	0.597	0.655	0.211	0.001
	Lilliefors	0.381	0.013	0.600	0.006	0.003	0.102	0.124	0.049	0.124	0.047	0.835	0.709	0.105	0.011
	Jarque-Bera	0.598	0.459	0.971	0.398	0.387	0.568	0.733	0.202	0.543	0.428	0.679	0.772	0.584	0.394
T2	Mean	13.17	12.98	13.68	13.33	12.01	11.90	14.76	13.96	15.29	19.14	11.28	16.72	14.98	13.83
	Standard deviation	0.22	0.44	0.27	0.63	0.43	0.85	0.71	0.84	0.34	1.30	0.77	0.50	0.38	0.56
Normality tests	Shapiro-Wilk	0.282	0.831	0.006	0.176	0.443	0.311	0.516	0.315	0.043	0.036	0.633	0.568	0.062	0.126
	Anderson-Darling	0.282	0.723	0.008	0.233	0.380	0.275	0.582	0.397	0.017	0.042	0.664	0.516	0.049	0.145
	Lilliefors	0.153	0.408	0.043	0.636	0.459	0.264	0.517	0.549	0.026	0.156	0.458	0.466	0.042	0.259
	Jarque-Bera	0.663	0.764	0.472	0.593	0.863	0.613	0.718	0.613	0.588	0.508	0.715	0.876	0.545	0.585
T3	Mean	15.63	17.81	13.55	12.90	14.02	13.63	15.06	15.60	18.69	14.48	13.86	17.64	15.13	15.21
	Standard deviation	0.37	0.35	0.45	0.33	1.12	0.73	0.40	0.82	0.65	0.50	0.47	0.46	0.40	0.91
Normality tests	Shapiro-Wilk	0.064	0.152	0.335	0.638	0.004	0.267	0.574	0.297	0.069	0.738	0.046	0.047	0.396	0.281
	Anderson-Darling	0.061	0.195	0.311	0.773	0.003	0.275	0.535	0.332	0.092	0.839	0.048	0.037	0.487	0.171
	Lilliefors	0.084	0.325	0.379	0.885	0.023	0.208	0.442	0.518	0.195	0.942	0.079	0.063	0.640	0.046
	Jarque-Bera	0.653	0.667	0.632	0.792	0.489	0.648	0.742	0.627	0.561	0.799	0.138	0.558	0.652	0.697
T4	Mean	10.93	12.86	9.46	9.48	11.46	10.37	13.38	13.45	13.62	14.82	11.11	13.10	11.60	13.53
	Standard deviation	0.44	0.73	0.15	0.82	1.04	1.04	0.98	0.35	0.32	0.49	0.45	0.32	0.52	0.58
Normality tests	0.314805	0.469	0.778	0.287	0.009	0.256	0.069	0.566	0.118	0.057	0.315	0.424	0.079	0.665	0.877
	0.186638	0.570	0.625	0.283	0.007	0.295	0.058	0.631	0.080	0.063	0.428	0.379	0.152	0.819	0.700
	0.25128	0.387	0.518	0.235	0.043	0.493	0.088	0.867	0.022	0.150	0.528	0.262	0.432	0.885	0.827
	0.909458	0.663	0.787	0.626	0.470	0.599	0.529	0.731	0.701	0.527	0.632	0.720	0.641	0.778	0.885
ANOVA	Contrast	Pr > Diff	Pr > Diff	Pr > Diff	Pr > Diff	Pr > Diff	Pr > Diff	Pr > Diff	Pr > Diff	Pr > Diff	Pr > Diff	Pr > Diff	Pr > Diff	Pr > Diff	Pr > Diff
	T1 vs T4	< 0.0001	< 0.0001	< 0.0001	< 0.0001	< 0.0001	< 0.0001	< 0.0001	< 0.0001	< 0.0001	< 0.0001	< 0.0001	< 0.0001	< 0.0001	< 0.0001
	T1 vs T2	< 0.0001	< 0.0001	< 0.0001	< 0.0001	< 0.0001	< 0.0001	< 0.0001	< 0.0001	< 0.0001	< 0.0001	< 0.0001	< 0.0001	< 0.0001	< 0.0001
	T1 vs T3	< 0.0001	< 0.0001	< 0.0001	< 0.0001	< 0.0001	< 0.0001	< 0.0001	< 0.0001	0.001	< 0.0001	< 0.0001	< 0.0001	< 0.0001	< 0.0001
	T3 vs T4	< 0.0001	< 0.0001	< 0.0001	< 0.0001	0.001456	< 0.0001	< 0.0001	< 0.0001	< 0.0001	< 0.0001	< 0.0001	< 0.0001	< 0.0001	0.000
	T3 vs T2	< 0.0001	< 0.0001	**0.74005**	**0.679195**	0.013389	< 0.0001	**0.709**	< 0.0001	< 0.0001	< 0.0001	< 0.0001	< 0.0001	**0.788**	0.001
	T2 vs T4	< 0.0001	**0.978783**	< 0.0001	< 0.0001	**0.795798**	0.000497	< 0.0001	**0.237**	< 0.0001	**0.803**	**0.866**	< 0.0001	< 0.0001	**0.820**

Supplementary Table 9. Effect of drought and salt stress on stomatal conductance rate in wheat plants. Gas exchange measurements were performed by a Licor 6400 gas analyzer under well watered (T1), water limited (T2), salt plus well watered (T3), and salt plus water limited (T4) conditions. Data (µmol CO_2 mol^{-1}) shown are the mean and SD (n=12-14 measurements). Calculation of mean and SD, tests for normal distribution of data, and one-way ANOVA analysis of the significance level between mean differences was performed by the XLSTAT software package. In the normality tests the p>0.05 values show normal distribution of data, while in the ANOVA table the p<0.05 show that the corresponding means are different at 5% significance level.

Treatmen	Cultivar	Tale 38	Azamatli 9	Giymatli 2	Gobustan	Gyrmyzy g	Balkan	NS 40S	NS Avanga	Subotican	Renesansa	Donnato	Midas	Gallio	Capo
T1	Mean	0.34	0.22	0.24	0.41	0.33	0.25	0.23	0.32	0.21	0.30	0.18	0.28	0.22	0.24
	Standard deviation	0.01	0.00	0.01	0.00	0.01	0.00	0.01	0.01	0.01	0.01	0.00	0.00	0.01	0.01
Normality tests	Shapiro-Wilk	0.009	0.208	0.029	0.269	0.803	0.873	0.888	0.519	0.398	0.026	0.576	0.071	0.226	0.059
	Anderson-Darling	0.012	0.225	0.025	0.307	0.513	0.769	0.843	0.424	0.414	0.029	0.670	0.100	0.234	0.052
	Lilliefors	0.052	0.332	0.085	0.429	0.529	0.723	0.841	0.153	0.495	0.047	0.926	0.143	0.179	0.053
	Jarque-Bera	0.359	0.459	0.493	0.697	0.964	0.861	0.823	0.700	0.741	0.334	0.753	0.608	0.436	0.532
T2	Mean	0.22	0.15	0.15	0.23	0.13	0.13	0.14	0.18	0.16	0.25	0.16	0.17	0.13	0.16
	Standard deviation	0.00	0.00	0.01	0.00	0.01	0.01	0.01	0.01	0.00	0.02	0.01	0.01	0.00	0.01
Normality tests	Shapiro-Wilk	0.589	0.108	0.857	0.021	0.693	0.705	0.584	0.437	0.594	0.003	0.038	0.998	0.150	0.342
	Anderson-Darling	0.505	0.124	0.794	0.027	0.447	0.469	0.709	0.395	0.711	0.003	0.049	0.982	0.153	0.180
	Lilliefors	0.646	0.355	0.856	0.103	0.384	0.516	0.755	0.312	0.739	0.020	0.129	0.935	0.532	0.149
	Jarque-Bera	0.680	0.585	0.808	0.158	0.906	0.917	0.723	0.678	0.766	0.522	0.566	0.886	0.639	0.704
T3	Mean	0.27	0.20	0.18	0.23	0.22	0.19	0.15	0.19	0.18	0.24	0.17	0.18	0.16	0.15
	Standard deviation	0.02	0.00	0.02	0.03	0.00	0.01	0.01	0.01	0.01	0.02	0.01	0.01	0.01	0.01
Normality tests	Shapiro-Wilk	0.611	0.203	0.073	0.597	0.114	0.054	0.036	0.630	0.676	0.069	0.900	0.916	0.081	0.618
	Anderson-Darling	0.552	0.184	0.053	0.463	0.111	0.047	0.039	0.724	0.495	0.063	0.892	0.789	0.094	0.756
	Lilliefors	0.337	0.153	0.056	0.192	0.110	0.063	0.045	0.757	0.216	0.099	0.698	0.840	0.215	0.935
	Jarque-Bera	0.713	0.644	0.475	0.696	0.618	0.566	0.257	0.740	0.823	0.577	0.776	0.916	0.588	0.743
T4	Mean	0.12	0.11	0.07	0.10	0.13	0.11	0.09	0.09	0.10	0.13	0.07	0.10	0.08	0.10
	Standard deviation	0.01	0.01	0.00	0.01	0.01	0.01	0.00	0.00	0.00	0.01	0.01	0.00	0.01	0.01
Normality tests	Shapiro-Wilk	0.611	0.203	0.073	0.597	0.114	0.054	0.036	0.630	0.676	0.069	0.900	0.916	0.081	0.618
	Anderson-Darling	0.552	0.184	0.053	0.463	0.111	0.047	0.039	0.724	0.495	0.063	0.892	0.789	0.094	0.756
	Lilliefors	0.337	0.153	0.056	0.192	0.110	0.063	0.045	0.757	0.216	0.099	0.698	0.840	0.215	0.935
	Jarque-Bera	0.713	0.644	0.475	0.696	0.618	0.566	0.257	0.740	0.823	0.577	0.776	0.916	0.588	0.743
ANOVA	Contrast	Pr > Diff	Pr > Diff	Pr > Diff	Pr > Diff	Pr > Diff	Pr > Diff	Pr > Diff	Pr > Diff	Pr > Diff	Pr > Diff	Pr > Diff	Pr > Diff	Pr > Diff	Pr > Diff
	T1 vs T4	< 0.0001	< 0.0001	< 0.0001	< 0.0001	< 0.0001	< 0.0001	< 0.0001	< 0.0001	< 0.0001	< 0.0001	< 0.0001	< 0.0001	< 0.0001	< 0.0001
	T1 vs T2	< 0.0001	< 0.0001	< 0.0001	< 0.0001	< 0.0001	< 0.0001	< 0.0001	< 0.0001	< 0.0001	< 0.0001	< 0.0001	< 0.0001	< 0.0001	< 0.0001
	T1 vs T3	< 0.0001	< 0.0001	< 0.0001	< 0.0001	< 0.0001	< 0.0001	< 0.0001	< 0.0001	< 0.0001	< 0.0001	0.003	< 0.0001	< 0.0001	< 0.0001
	T3 vs T4	< 0.0001	< 0.0001	< 0.0001	< 0.0001	< 0.0001	< 0.0001	< 0.0001	< 0.0001	< 0.0001	< 0.0001	< 0.0001	< 0.0001	< 0.0001	< 0.0001
	T3 vs T2	< 0.0001	< 0.0001	< 0.0001	**0.705**	< 0.0001	< 0.0001	0.000	0.003	< 0.0001	**0.232**	0.003	0.000	< 0.0001	0.036
	T2 vs T4	< 0.0001	< 0.0001	< 0.0001	< 0.0001	**0.998**	0.000	< 0.0001	< 0.0001	< 0.0001	< 0.0001	< 0.0001	< 0.0001	< 0.0001	< 0.0001

Supplementary Table 10. Effect of drought and salt stress on internal CO_2 concentration in wheat plants. Gas exchange measurements were performed by a Licor 6400 gas analyzer under well watered (T1), water limited (T2), salt plus well watered (T3), and salt plus water limited (T4) conditions. Data (mol H_2O m^{-2} s^{-1}) shown are the mean and SD (n=12-14 measurements). Calculation of mean and SD, tests for normal distribution of data, and one-way ANOVA analysis of the significance level between mean differences was performed by the XLSTAT software package. In the normality tests the p>0.05 values show normal distribution of data, while in the ANOVA table the p<0.05 show that the corresponding means are different at 5% significance level.

Treatmen	Cultivar	Tale 38	Azamatli 9	Giymatli 2	Gobustan	Gyrmyzy g	Balkan	NS 40S	NS Avanga	Subotican	Renesansa	Donnato	Midas	Gallio	Capo
T1	Mean	252.15	251.31	235.33	253.54	259.77	256.62	260.69	282.92	224.77	260.62	250.08	280.85	239.00	250.31
	Standard deviation	7.06	2.87	6.68	7.30	5.90	2.75	4.39	8.09	2.55	3.91	3.84	5.19	11.90	4.33
Normality tests	Shapiro-Wilk	0.013	0.448	0.020	0.011	0.181	0.012	0.036	0.221	0.657	0.220	0.045	0.130	0.048	0.986
	Anderson-Darling	0.006	0.400	0.012	0.003	0.148	0.006	0.029	0.173	0.470	0.256	0.078	0.220	0.050	0.911
	Lilliefors	0.006	0.418	0.006	0.007	0.146	0.029	0.007	0.276	0.347	0.480	0.319	0.550	0.261	0.915
	Jarque-Bera	0.435	0.496	0.467	0.447	0.544	0.417	0.518	0.604	0.752	0.328	0.518	0.261	0.484	0.840
T2	Mean	221.58	235.00	226.00	211.42	212.58	218.08	216.08	239.42	208.67	255.08	232.67	226.67	248.38	219.75
	Standard deviation	5.25	3.79	3.07	2.68	9.47	9.65	4.89	3.73	6.50	3.20	4.08	7.60	7.07	10.35
Normality tests	Shapiro-Wilk	0.554	0.870	0.082	0.013	0.192	0.140	0.038	0.393	0.131	0.933	0.764	0.886	0.017	0.012
	Anderson-Darling	0.536	0.836	0.067	0.023	0.146	0.119	0.047	0.362	0.111	0.942	0.604	0.884	0.032	0.014
	Lilliefors	0.424	0.851	0.082	0.094	0.236	0.184	0.074	0.488	0.027	0.960	0.551	0.918	0.232	0.094
	Jarque-Bera	0.770	0.838	0.689	0.210	0.672	0.611	0.144	0.687	0.602	0.844	0.804	0.880	0.204	0.545
T3	Mean	229.83	256.67	234.42	249.75	243.00	248.00	243.75	274.92	234.67	260.58	237.83	262.25	257.25	261.75
	Standard deviation	5.56	5.97	8.67	8.40	5.58	9.73	6.47	7.10	2.29	6.16	9.10	8.54	7.94	2.86
Normality tests	Shapiro-Wilk	229.833	256.667	234.417	249.750	243.000	248.000	243.750	274.917	234.667	260.583	237.833	262.250	257.250	261.750
	Anderson-Darling	5.557	5.975	8.670	8.400	5.576	9.733	6.468	7.103	2.291	6.156	9.104	8.540	7.944	2.864
	Lilliefors	0.611	0.203	0.073	0.597	0.114	0.054	0.036	0.630	0.676	0.069	0.900	0.916	0.081	0.618
	Jarque-Bera	0.552	0.184	0.053	0.463	0.111	0.047	0.039	0.724	0.495	0.063	0.892	0.789	0.094	0.756
T4	Mean	215.42	175.00	155.33	180.25	142.10	122.75	162.58	145.67	172.92	157.58	149.36	149.00	141.45	164.40
	Standard deviation	10.96	3.02	13.30	22.23	9.42	14.78	20.17	8.62	16.34	13.01	26.10	10.17	20.69	6.95
Normality tests	Shapiro-Wilk	0.168	0.421	0.011	0.024	0.822	0.530	0.074	0.248	0.007	0.072	0.001	0.145	0.260	0.844
	Anderson-Darling	0.060	0.295	0.009	0.025	0.850	0.614	0.049	0.179	0.006	0.099	0.002	0.127	0.347	0.803
	Lilliefors	0.017	0.346	0.005	0.012	0.849	0.643	0.011	0.230	0.008	0.149	0.009	0.029	0.580	0.586
	Jarque-Bera	0.875	0.690	0.544	0.531	0.750	0.770	0.586	0.657	0.527	0.609	0.514	0.603	0.667	0.860
ANOVA	Contrast	Pr > Diff	Pr > Diff	Pr > Diff	Pr > Diff	Pr > Diff	Pr > Diff	Pr > Diff	Pr > Diff	Pr > Diff	Pr > Diff	Pr > Diff	Pr > Diff	Pr > Diff	Pr > Diff
	T1 vs T4	< 0.0001	< 0.0001	< 0.0001	< 0.0001	< 0.0001	< 0.0001	< 0.0001	< 0.0001	< 0.0001	< 0.0001	< 0.0001	< 0.0001	< 0.0001	< 0.0001
	T1 vs T2	< 0.0001	< 0.0001	**0.057**	< 0.0001	< 0.0001	< 0.0001	< 0.0001	< 0.0001	< 0.0001	**0.274**	0.013	< 0.0001	0.007	< 0.0001
	T1 vs T3	< 0.0001	0.010	**0.994**	**0.871**	< 0.0001	**0.157**	0.002	0.037	**0.076**	**1.000**	**0.124**	< 0.0001	**0.466**	0.001
	T3 vs T4	0.000	< 0.0001	< 0.0001	< 0.0001	< 0.0001	< 0.0001	< 0.0001	< 0.0001	< 0.0001	< 0.0001	< 0.0001	< 0.0001	< 0.0001	< 0.0001
	T3 vs T2	0.049	< 0.0001	**0.101**	< 0.0001	< 0.0001	< 0.0001	< 0.0001	< 0.0001	0.000	**0.295**	**0.787**	< 0.0001	**0.404**	< 0.0001
	T2 vs T4	0.202	< 0.0001	< 0.0001	< 0.0001	< 0.0001	< 0.0001	< 0.0001	< 0.0001	< 0.0001	< 0.0001	< 0.0001	< 0.0001	< 0.0001	< 0.0001

Supplementary Table 11. Effect of drought and salt stress on evaporation in wheat plants. Gas exchange measurements were performed by a Licor 6400 gas analyzer under well watered (T1), water limited (T2), salt plus well watered (T3), and salt plus water limited (T4) conditions. Data (mmol H_2O $m^{-2}s^{-1}$) shown are the mean and SD (n=12-14 measurements). Calculation of mean and SD, tests for normal distribution of data, and one-way ANOVA analysis of the significance level between mean differences was performed by the XLSTAT software package. In the normality tests the p>0.05 values show normal distribution of data, while in the ANOVA table the p<0.05 show that the corresponding means are different at 5% significance level.

Treatmen	Cultivar	Tale 38	Azamatli 9	Giymatli 2	Gobustan	Gyrmyzy g	Balkan	NS 40S	NS Avanga	Subotican	Renesansa	Donnato	Midas	Gallio	Capo
T1	Mean	5.67	4.07	5.10	5.99	5.19	5.12	3.78	4.34	3.29	4.21	3.85	4.98	3.98	4.59
	Standard deviation	0.48	0.06	0.73	0.20	0.32	0.37	0.28	0.20	0.14	0.14	0.30	0.48	0.21	0.26
Normality tests	Shapiro-Wilk	0.004	0.022	0.001	0.092	0.007	0.023	0.011	0.221	0.066	0.039	0.020	0.014	0.015	0.023
	Anderson-Darling	0.003	0.021	0.001	0.057	0.006	0.024	0.008	0.264	0.070	0.050	0.020	0.014	0.015	0.027
	Lilliefors	0.004	0.092	0.006	0.086	0.032	0.099	0.009	0.162	0.091	0.162	0.036	0.042	0.093	0.157
	Jarque-Bera	0.489	0.453	0.475	0.581	0.496	0.526	0.515	0.652	0.531	0.548	0.514	0.509	0.522	0.525
T2	Mean	4.14	3.07	3.15	4.18	2.45	2.60	2.61	3.23	2.63	3.21	2.28	2.66	1.93	3.17
	Standard deviation	0.24	0.08	0.09	0.14	0.20	0.21	0.33	0.11	0.04	0.04	0.12	0.50	0.10	0.10
Normality tests	Shapiro-Wilk	0.01	0.63	0.98	0.10	0.04	0.35	0.00	0.36	0.32	0.49	0.08	0.01	0.06	0.67
	Anderson-Darling	0.00	0.68	0.89	0.12	0.05	0.19	0.00	0.39	0.35	0.55	0.08	0.01	0.06	0.77
	Lilliefors	0.01	0.86	0.87	0.11	0.04	0.10	0.01	0.45	0.39	0.88	0.05	0.01	0.08	0.88
	Jarque-Bera	0.49	0.73	0.97	0.61	0.54	0.65	0.49	0.65	0.66	0.68	0.54	0.50	0.39	0.71
T3	Mean	3.85	3.59	3.48	4.60	4.13	3.10	2.32	3.68	3.15	3.27	2.65	2.78	3.34	3.60
	Standard deviation	0.08	0.23	0.26	0.20	0.19	0.09	0.12	0.15	0.06	0.18	0.24	0.18	0.28	0.16
Normality tests	Shapiro-Wilk	0.780	0.009	0.022	0.006	0.024	0.938	0.933	0.271	0.134	0.018	0.002	0.612	0.857	0.048
	Anderson-Darling	0.583	0.008	0.028	0.004	0.020	0.936	0.889	0.339	0.129	0.016	0.001	0.659	0.917	0.042
	Lilliefors	0.591	0.005	0.026	0.022	0.034	0.720	0.611	0.356	0.034	0.036	0.007	0.679	0.984	0.014
	Jarque-Bera	0.796	0.466	0.504	0.440	0.486	0.782	0.811	0.600	0.531	0.475	0.421	0.659	0.778	0.409
T4	Mean	2.97	2.35	1.96	2.29	2.92	2.55	2.07	1.74	1.94	2.25	1.42	1.97	1.79	1.68
	Standard deviation	0.13	0.06	0.06	0.21	0.14	0.14	0.11	0.12	0.04	0.10	0.11	0.42	0.14	0.12
Normality tests	Shapiro-Wilk	0.476	0.784	0.625	0.269	0.700	0.170	0.680	0.030	0.910	0.150	0.016	0.001	0.020	0.001
	Anderson-Darling	0.509	0.713	0.780	0.155	0.779	0.111	0.642	0.027	0.665	0.156	0.013	0.001	0.020	0.000
	Lilliefors	0.543	0.440	0.908	0.111	0.724	0.139	0.528	0.012	0.541	0.217	0.007	0.001	0.004	0.001
	Jarque-Bera	0.673	0.748	0.684	0.626	0.708	0.527	0.693	0.519	0.997	0.552	0.335	0.414	0.276	0.155
ANOVA	Contrast	Pr > Diff	Pr > Diff	Pr > Diff	Pr > Diff	Pr > Diff	Pr > Diff	Pr > Diff	Pr > Diff	Pr > Diff	Pr > Diff	Pr > Diff	Pr > Diff	Pr > Diff	Pr > Diff
	T1 vs T4	< 0.0001	< 0.0001	< 0.0001	< 0.0001	< 0.0001	< 0.0001	< 0.0001	< 0.0001	< 0.0001	< 0.0001	< 0.0001	< 0.0001	< 0.0001	< 0.0001
	T1 vs T2	< 0.0001	< 0.0001	< 0.0001	< 0.0001	< 0.0001	< 0.0001	< 0.0001	< 0.0001	< 0.0001	< 0.0001	< 0.0001	< 0.0001	< 0.0001	< 0.0001
	T1 vs T3	< 0.0001	< 0.0001	< 0.0001	< 0.0001	< 0.0001	< 0.0001	< 0.0001	< 0.0001	0.002	< 0.0001	< 0.0001	< 0.0001	< 0.0001	< 0.0001
	T3 vs T4	< 0.0001	< 0.0001	< 0.0001	< 0.0001	< 0.0001	< 0.0001	< 0.0001	< 0.0001	< 0.0001	< 0.0001	< 0.0001	0.000	< 0.0001	< 0.0001
	T3 vs T2	**0.059**	< 0.0001	**0.210**	< 0.0001	< 0.0001	< 0.0001	0.021	< 0.0001	< 0.0001	**0.593**	0.001	**0.923**	< 0.0001	< 0.0001
	T2 vs T4	< 0.0001	< 0.0001	< 0.0001	< 0.0001	< 0.0001	**0.936**	0.046	< 0.0001	< 0.0001	< 0.0001	< 0.0001	0.002	**0.309**	< 0.0001

Supplementary Table 12. Proline content (mg g^{-1} fresh weight) of wheat leaves. Measurements were performed under well watered (T1), water limited (T2), salt plus well watered (T3), and salt plus water limited (T4) conditions. Data shown are mean± SE (n=3-5) plants/treatment. Calculation of mean and SD, tests for normal distribution of data, and one-way ANOVA analysis of the significance level between mean differences was performed by the XLSTAT software package. In the normality tests the p>0.05 values show normal distribution of data, while in the ANOVA table the p<0.05 show that the corresponding means are different at 5% significance level.

Treatmen	Cultivar	Tale 38	Azamatli	Giymatli 2	Gobustan	Gyrmyzy	Balkan	NS 40S	NS Avang	Suboticar	Renesans	Donnato	Midas	Gallio	Capo
T1	Mean	13.333	0.051	12.333	11.933	11.200	9.867	0.054	10.933	0.050	9.120	12.960	17.000	16.840	12.400
	Standard deviation	0.757	0.006	2.318	0.643	4.067	2.003	0.009	2.053	0.007	1.361	6.752	6.274	5.222	0.917
Normality tests	Shapiro-Wilk	0.253	0.298	0.165	0.298	0.661	0.890	0.554	0.567	0.583	0.806	0.134	0.420	0.277	0.637
	Anderson-Darling	0.169	0.200	0.118	0.200	0.505	0.613	0.415	0.426	0.440	0.578	0.105	0.296	0.185	0.487
	Lilliefors	0.196	0.230	0.144	0.230	0.663	0.929	0.512	0.529	0.552	0.852	0.129	0.345	0.213	0.628
	Jarque-Bera	0.781	0.787	0.773	0.787	0.841	0.866	0.825	0.827	0.829	0.859	0.771	0.804	0.784	0.838
T2	Mean	18.550	19.800	20.250	18.560	12.267	14.867	0.056	16.133	0.055	16.760	11.300	12.840	24.550	16.700
	Standard deviation	3.682	3.265	5.382	4.586	2.139	1.665	0.013	2.203	0.003	2.964	0.500	3.629	13.369	6.368
Normality tests	Shapiro-Wilk	0.475	0.127	0.447	0.177	0.089	0.463	0.503	0.174	0.780	0.859	1.000	0.417	**0.025**	0.456
	Anderson-Darling	0.344	0.102	0.319	0.124	0.086	0.334	0.369	0.122	0.565	0.602	0.631	0.294	0.064	0.327
	Lilliefors	0.409	0.126	0.376	0.150	0.110	0.395	0.444	0.148	0.822	0.904	0.974	0.342	0.088	0.386
	Jarque-Bera	0.812	0.771	0.808	0.774	0.769	0.810	0.817	0.774	0.856	0.864	0.869	0.803	0.767	0.809
T3	Mean	12.533	8.800	8.467	11.933	13.840	9.200	0.056	12.800	0.046	8.667	10.667	16.600	10.960	8.667
	Standard deviation	0.115	1.732	1.286	2.386	5.816	1.929	0.010	2.946	0.005	1.270	2.468	5.481	2.233	0.462
Normality tests	Shapiro-Wilk	**< 0.0001**	**< 0.0001**	0.298	0.485	**0.026**	0.298	0.942	0.065	0.878	**< 0.0001**	0.549	0.386	0.683	**< 0.0001**
	Anderson-Darling	0.057	0.057	0.200	0.353	0.064	0.200	0.626	0.077	0.609	0.057	0.410	0.268	0.514	0.057
	Lilliefors	0.081	0.081	0.230	0.422	0.088	0.230	0.962	0.101	0.920	0.081	0.505	0.309	0.694	0.081
	Jarque-Bera	0.767	0.767	0.787	0.814	0.767	0.787	0.868	0.768	0.865	0.767	0.824	0.798	0.844	0.767
T4	Mean	29.667	23.600	38.050	37.000	42.240	46.000	0.123	43.867	0.181	54.250	14.850	36.000	67.800	19.400
	Standard deviation	3.301	8.371	11.234	6.602	7.718	16.373	0.027	9.159	0.041	16.058	3.117	21.086	6.239	4.297
Normality tests	Shapiro-Wilk	0.527	0.271	0.261	**< 0.0001**	0.866	0.848	0.253	0.463	0.399	0.526	0.100	0.685	0.787	0.806
	Anderson-Darling	0.391	0.181	0.175	0.057	0.605	0.598	0.169	0.334	0.278	0.390	0.091	0.515	0.569	0.578
	Lilliefors	0.476	0.209	0.202	0.081	0.910	0.894	0.196	0.395	0.323	0.475	0.114	0.697	0.831	0.852
	Jarque-Bera	0.821	0.783	0.782	0.767	0.864	0.863	0.781	0.810	0.800	0.820	0.769	0.845	0.857	0.859
ANOVA	Contrast	Pr > Diff	Pr > Diff	Pr > Diff	Pr > Diff	Pr > Diff	Pr > Diff	Pr > Diff	Pr > Diff	Pr > Diff	Pr > Diff	Pr > Diff	Pr > Diff	Pr > Diff	Pr > Diff
	T1 vs T4	0.114	0.001	0.474	0.274	0.994	0.917	0.998	0.608	0.991	0.453	0.974	0.978	0.454	0.308
	T1 vs T2	0.982	0.181	0.901	1.000	0.881	1.000	0.997	0.967	0.995	1.000	0.908	0.906	0.620	0.003
	T1 vs T3	0.064	0.043	0.181	0.274	0.980	0.885	1.000	0.848	0.950	0.523	0.999	0.994	0.078	0.087
	T3 vs T4	0.000	0.000	0.003	0.000	0.000	0.004	0.003	0.000	0.000	0.000	0.931	0.387	0.000	0.035
	T3 vs T2	0.002	0.658	0.020	0.000	0.000	0.009	0.004	0.001	0.000	0.000	0.822	0.266	0.000	0.471
	T2 vs T4	0.000	0.010	0.001	0.000	0.000	0.003	0.004	0.000	0.000	0.000	0.665	0.175	0.000	0.003
